# Gene regulatory networks for lignin biosynthesis in switchgrass *(Panicum virgatum*)

**DOI:** 10.1111/pbi.13000

**Published:** 2018-09-17

**Authors:** Xiaolan Rao, Xin Chen, Hui Shen, Qin Ma, Guifen Li, Yuhong Tang, Maria Pena, William York, Taylor P. Frazier, Scott Lenaghan, Xirong Xiao, Fang Chen, Richard A. Dixon

**Affiliations:** ^1^ BioDiscovery Institute and Department of Biological Sciences University of North Texas Denton TX USA; ^2^ BioEnergy Science Center (BESC) Oak Ridge National Laboratory Oak Ridge TN USA; ^3^ Center for Applied Mathematics Tianjin University Tianjin China; ^4^ Department of Agronomy, Horticulture, and Plant Science and Department of Mathematics and Statistics South Dakota State University Brookings SD USA; ^5^ Noble Research Institute Ardmore OK USA; ^6^ Complex Carbohydrate Research Center and Department of Biochemistry and Molecular Biology University of Georgia Athens GA USA; ^7^ Department of Plant Sciences University of Tennessee Knoxville TN USA; ^8^ Department of Food Science University of Tennessee Knoxville TN USA; ^9^ Center for Bioenergy Innovation (CBI) Oak Ridge National Laboratory Oak Ridge TN USA; ^10^ Present address: Marker‐assisted Breeding and Traits Chromatin Inc Lubbock TX 79404 USA

**Keywords:** Bi‐clustering algorithm, bioenergy crop, co‐expression analysis, transcription factors, transgenic switchgrass, secondary cell wall biosynthesis

## Abstract

Cell wall recalcitrance is the major challenge to improving saccharification efficiency in converting lignocellulose into biofuels. However, information regarding the transcriptional regulation of secondary cell wall biogenesis remains poor in switchgrass (*Panicum virgatum*), which has been selected as a biofuel crop in the United States. In this study, we present a combination of computational and experimental approaches to develop gene regulatory networks for lignin formation in switchgrass. To screen transcription factors (TFs) involved in lignin biosynthesis, we developed a modified method to perform co‐expression network analysis using 14 lignin biosynthesis genes as bait (target) genes. The switchgrass lignin co‐expression network was further extended by adding 14 TFs identified in this study, and seven TFs identified in previous studies, as bait genes. Six TFs (PvMYB58/63, PvMYB42/85, PvMYB4, PvWRKY12, PvSND2 and PvSWN2) were targeted to generate overexpressing and/or down‐regulated transgenic switchgrass lines. The alteration of lignin content, cell wall composition and/or plant growth in the transgenic plants supported the role of the TFs in controlling secondary wall formation. RNA‐seq analysis of four of the transgenic switchgrass lines revealed downstream target genes of the secondary wall‐related TFs and crosstalk with other biological pathways. *In vitro* transactivation assays further confirmed the regulation of specific lignin pathway genes by four of the TFs. Our meta‐analysis provides a hierarchical network of TFs and their potential target genes for future manipulation of secondary cell wall formation for lignin modification in switchgrass.

## Introduction

The cell wall is deposited outside the plant cell membrane as a cellular exoskeleton that can be classified as primary or secondary depending on function and composition (Vogel, 2008). The secondary cell wall polymers cellulose, hemicellulose and lignin constitute the most abundant source of plant biomass that provides raw materials for generating renewable biofuels (Bouton, 2007; Pauly and Keegstra, 2010). However, plant cell walls possess chemical and structural properties (called ‘biomass recalcitrance’) to help prevent microbial and enzymatic deconstruction (Himmel *et al*., 2007). Overcoming biomass recalcitrance is a major challenge in the lignocellulosic‐bioenergy industry. Lignin, covalently incorporated into the cross‐linked matrix of polysaccharides, prevents access of hydrolytic enzymes for degrading the lignocellulosic components to monosaccharides for subsequent biofuel production (Pauly and Keegstra, 2010). Conversely, lignin has potential to be developed as a high value co‐product of bioprocessing (Ragauskas *et al*., 2014). For both applications, knowledge of the gene regulatory networks underlying lignin and secondary cell wall biosynthesis is necessary to facilitate targeted genetic interventions.

Switchgrass (*Panicum virgatum* L.) has been selected as a major cellulosic feedstock for bioconversion to ethanol in the United States (Bouton, 2007). Manipulating cell walls in switchgrass through down‐regulation of GAUT4 (*galacturonosyltransferase4,* involved in pectin biosynthesis), COMT (*caffeic acid/5‐hydroxyferulic acid 3‐O‐methyltransferase,* involved in lignin biosynthesis) and FPGS (*folylpolyglutamate synthase 1,* involved in C1 metabolism) or overexpression of MYB4 (a repressor of lignin biosynthesis) in all cases leads to a reduction of cell wall recalcitrance and an increased efficiency of sugar release (Dumitrache *et al*., 2017). However, as a non‐model organism, the lack of genetic information on secondary cell wall formation in switchgrass limits biotechnological approaches to feedstock development. Previously we have identified genes involved in monolignol biosynthesis and provided candidate structural genes associated with secondary wall development in brassinosteroid‐induced switchgrass suspension cultures (Rao *et al*., 2017; Shen *et al*., 2013). Besides characterizing metabolic genes in secondary wall biosynthesis, understanding the transcription factors (TFs) that regulate secondary wall formation is required to enable rational biodesign of switchgrass as a bioenergy crop.

In recent decades, studies on *Arabidopsis thaliana* have contributed to a thorough analysis of TFs involved in secondary wall regulation, including sub‐group members of the NAC, MYB, WRKY and other TF families (Nakano *et al*., 2015; Taylor‐Teeples *et al*., 2015; Wang and Dixon, 2012; Zhong and Ye, 2015). A hierarchical organization is observed in the transcriptional regulatory system in Arabidopsis that regulates secondary wall biosynthesis in concert with other metabolic pathways. However, few TFs have been reported that control secondary wall development in grasses, especially in switchgrass (Rao and Dixon, 2018; Shen *et al*., 2012; Zhong *et al*., 2015). Switchgrass (Pv) SWNs and PvMYB46 have been shown to be activators of secondary wall biosynthesis based on ectopic expression in Arabidopsis (Zhong *et al*., 2015). PvSWNs and PvMYB46 are capable of rescuing the secondary wall defects in the Arabidopsis *snd1*/*nst1* and *myb46*/*myb83* double mutants, respectively, and overexpression of PvSWNs and PvMYB46 in Arabidopsis leads to activation of the secondary wall biosynthesis programme (Zhong *et al*., 2015). We have characterized PvMYB4 as a lignin repressor; overexpression of PvMYB4 in transgenic switchgrass and tobacco results in the down‐regulation of lignin biosynthesis genes and a reduced lignin content (Shen *et al*., 2012). Other TFs involved in switchgrass secondary wall regulation remain largely unexplored.

With the increased number of public microarray data sets, a co‐expression approach has been widely used to investigate TF candidates and their potential target genes because the expression of transcriptional regulators and their targets tends to be coordinated (Ruprecht and Persson, 2012; Serin *et al*., 2016). Several groups have applied a combination of large‐scale co‐expression analysis and experimental evaluation to discover novel TFs involved in cell wall formation in Arabidopsis, rice, maize and sugarcane (Cassan‐Wang *et al*., 2013; Ferreira *et al*., 2016; Hansen *et al*., 2014; Hirano *et al*., 2013a; Ruprecht and Persson, 2012; Ruprecht *et al*., 2011).

To understand the transcriptional network regulating lignin biosynthesis in switchgrass, we here present a computational approach with co‐expression and RNA‐seq analyses to explore genes that are strongly associated with validated TFs, complemented by *in planta* validation by transgenesis. First, we developed a new method based on a bi‐clustering algorithm to detect TFs that are strongly co‐expressed with previously validated lignin biosynthesis genes in switchgrass, and extended the switchgrass co‐expression network by adding 21 switchgrass TF genes identified as secondary wall regulators as seed genes. Second, we targeted six TFs (PvMYB58/63, PvMYB42/85, PvMYB4, PvWRKY12, PvSND2 and PvSWN2) for overexpression and/or down‐regulation in transgenic switchgrass, and analysed the consequences by RNA‐seq analysis. Finally, interactions between TFs and their candidate target promoters were interrogated by *in vitro* transactivation assays. Together these analyses revealed secondary wall‐ related TFs, their downstream target genes, and the crosstalk between secondary wall‐related TFs and other biological pathways, to inform targeted modification of cell wall composition for enhancing the processing of switchgrass biomass.

## Results

### Co‐expression screening of switchgrass TFs involved in lignin biosynthesis

To detect genes with similar expression patterns in large data sets, we developed a modified method for co‐expression analysis based on QUBIC, a previously reported bi‐clustering algorithm (Li *et al*., 2009; Zhang *et al*., 2017). This method is effective in identifying co‐expression relationships in gene pairs under both ‘all’ and ‘some’ (to‐be‐identified) conditions. A detailed description of the approach, along with a rationale and comparison with the classical Pearson correlation method, is provided in Methods S1.

To identify candidate TFs involved in secondary cell wall biosynthesis, we performed a comparative co‐expression analysis with a large scale data set of Arabidopsis and switchgrass transcriptomes using lignin biosynthesis genes as target (bait) genes. Specifically, 16 and 14 lignin biosynthesis genes were used as baits to search genes with correlated expression pattern against Arabidopsis (GSE34188, 99 samples) (Hanada *et al*., 2013) and switchgrass (93 samples) (Zhang *et al*., 2013) public microarray data sets respectively (Table S1). The bait genes represented all 11 families of enzymes responsible for monolignol biosynthesis (Shen *et al*., 2013). The selected microarray data sets in Arabidopsis and switchgrass represented similar conditions including major tissue and organ types during developmental stages of the entire plant life‐cycle, and mature plants exposed to a series of stress treatments. The co‐expressed genes were determined as described in Methods S1. Using the approach, we identified 645 and 1274 transcription factors coordinated with 16 and 14 lignin biosynthesis genes in Arabidopsis and switchgrass respectively (Dataset S1).

**Figure 1:**
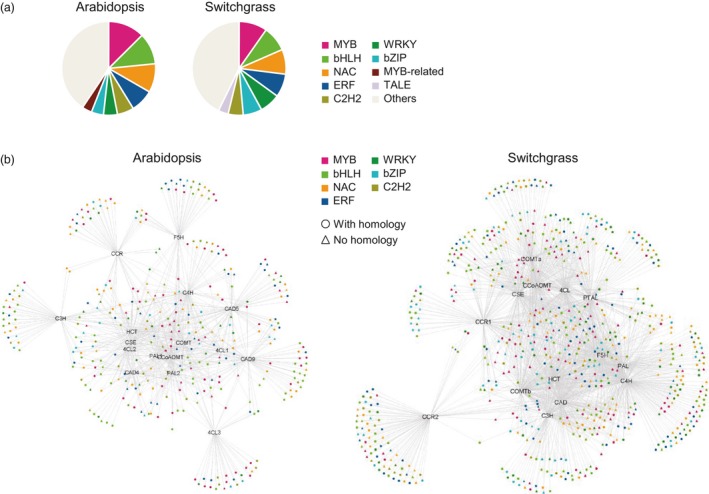
Co‐expression networks for lignin biosynthesis in Arabidopsis and switchgrass. (a) Distribution of TF families that are co‐expressed with lignin biosynthesis genes. (b) Selected TF families in the co‐expression network of lignin biosynthesis. Colours represent TF families. Circles and triangles represent genes with and without homologs, respectively, appearing in both the Arabidopsis and switchgrass networks.

In both species, TFs in the co‐expression network with lignin biosynthesis genes displayed a similar distribution of TF families (Figure 1a and Table S2). They shared the same categories of the four most abundant families sorted in descending order; MYB (81 in Arabidopsis and 124 in switchgrass), bHLH (70 in Arabidopsis and 111 in switchgrass), NAC (64 in Arabidopsis and 109 in switchgrass) and ERF (51 in Arabidopsis and 102 in switchgrass). The C2H2‐, WRKY‐ and bZIP‐type TFs were subsequently listed in the fifth to seventh places in both Arabidopsis and switchgrass. To obtain phylogenetic relationships of the secondary wall‐associated TFs, we generated phylogenetic trees of the MYB, NAC, bHLH, ERF and WRKY‐type TFs that appeared in the co‐expression networks (Figure S1). The analysis showed that many of the Arabidopsis and switchgrass genes were grouped into the same clades according to their protein sequences (Figure S1). We next used the BLASTp program to search switchgrass TF proteins against Arabidopsis TF proteins in the network. This revealed that 522 switchgrass TFs have homologs that were present in the Arabidopsis network, corresponding to 202 Arabidopsis TFs (Dataset S1). Many of the homologs tended to be co‐expressed with similar lignin biosynthesis genes in the two species (Figure 1b). Some have previously been shown to be involved in secondary cell wall regulation in Arabidopsis and/or switchgrass. For example, the expression of *AtMYB46* (AT5G12870) and its homolog *PvMYB46* (KanlowCTG44101_s_at) is tightly correlated with expression of *PAL*,* 4CL*,* COMT* and *CCoAoMT*, but not with *C3′H* and *HCT*, in both species (Dataset S1). P*vMYB46* is a functional ortholog of Arabidopsis *MYB46,* with ability to rescue cell wall defects in the Arabidopsis *myb46*/*myb83* double mutant (Zhong *et al*., 2015).

The above observations suggest that Arabidopsis and switchgrass have evolved a conserved mechanism of secondary cell wall biosynthesis by recruiting homologous members of certain TF families. However, a close observation revealed different features in the TF co‐expression networks between the two species (Figure S2). For example, Arabidopsis *F5H* shared a smaller proportion of co‐expressed TFs with other lignin biosynthesis genes (38 out of 78 TFs) than did switchgrass *F5H* (238 of 259 TFs). *AtMYB58* and *AtMYB63* are co‐expressed with most lignin biosynthesis genes except *F5H* in Arabidopsis (Figure S2). This is consistent with their role in activating the expression of lignin biosynthesis genes via binding to AC elements in the target gene's promoter region with exclusion of F5H, the promoter of which does not contain AC‐rich elements (Zhao and Dixon, 2011; Zhou *et al*., 2009). However, the switchgrass homologs of *MYB58/63* (AP13ITG56055_at and AP13ITG57154RC_at) showed a correlated expression with *F5H* and most other lignin biosynthesis genes (Figure S2), suggesting that *PvMYB58/63* may be involved in the regulation of *F5H* expression in switchgrass, but not in Arabidopsis.

### Functional analysis of TF candidates by transgenesis

To evaluate the TF candidates identified in the co‐expression network, we selected six genes belonging to the MYB, NAC and WRKY families for overexpression and/or down‐regulation in transgenic switchgrass. These genes were selected from the co‐expression network based on the number of correlated genes and the strength of the correlation between gene pairs. *PvMYB58/63A* (AP13ITG56055_at) is co‐expressed with 12 lignin biosynthesis genes with the highest BF value of 81. *PvMYB42/85A* (AP13CTG22878_at) is co‐expressed with 10 lignin biosynthesis genes with the highest BF value of 72. *PvSWN2A* and *PvSWB2B* are co‐expressed with 6 and 8 lignin biosynthesis genes with the highest BF values of 69 and 55 respectively. *PvSND2* is co‐expressed with 10 lignin biosynthesis genes with the highest BF value of 93. *PvMYB4* is co‐expressed with 2 lignin biosynthesis genes and has been identified as negative regulator in our previous publication (Shen *et al*., 2012), and the phenotype of the PvMYB4‐OX line has been described in a previous report (Shen *et al*., 2012). *PvWRKY12* shows co‐expression with *PvSWN1* and *PvSWN2*, similar to its homolog *AtWRKY12* in the Arabidopsis co‐expression network. We selected *PvWRKY12* as a candidate to explore potential regulation by PvSWN1 and PvSWN2.

#### PvMYB58/63

**Figure 2:**
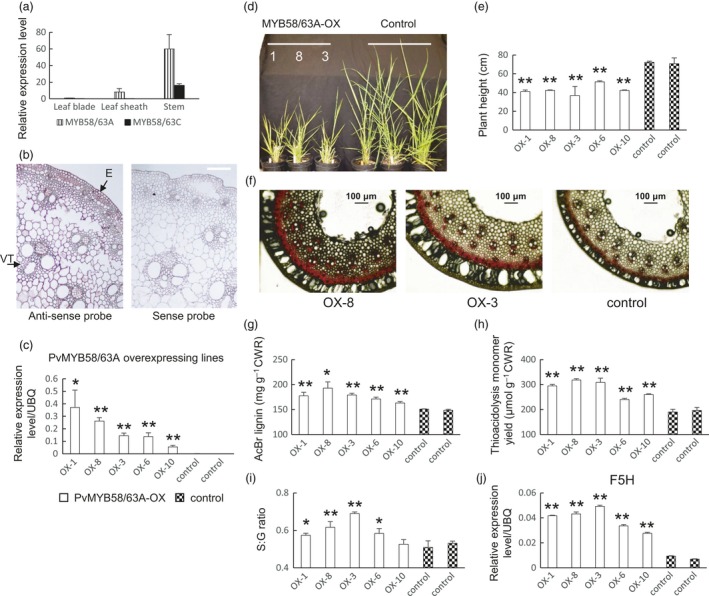
Phenotype of transgenic switchgrass overexpressing PvMYB58/63A. (a) qRT‐PCR analysis of PvMYB58/63A and PvMYB58/63C transcript levels in leaf blade, leaf sheath and stem. The transcript level in leaf blade was set to 1. (b) *In situ* hybridization of PvMYB58/63A in switchgrass stem, E4 stage, internode 2. VT, vascular tissue; E, epidermis. Bars, 100 μm. (c) qRT‐PCR analysis of PvMYB58/63A transcripts in control and PvMYB58/63A‐OX transgenic switchgrass. UBQ, ubiquitin. (d) Representative photograph of control and PvMYB58/63A‐OX lines. (e) Plant height for control and PvMYB58/63A‐OX lines. (f) Phloroglucinol‐HCl staining of stem cross sections (internode 2 at the E2 stage) of PvMYB58/63A‐OX transgenic switchgrass. Bars, 100 μm. (g) Total lignin content of whole tillers at R1 stage determined by the AcBr method. (h) Total lignin content and (i) S/G ratio determined by thioacidolysis. (j) qRT‐PCR analysis of ferulate 5‐hydroxylase (F5H) transcripts in control and PvMYB58/63A‐OX transgenic switchgrass. All data are means ± SE (*n* = 3). Significant differences from the equivalent control were determined by the Student's *t*‐test and are represented by a single asterisk (*P* < 0.05) or double asterisk (*P* < 0.01).

Four genes in switchgrass clustered in the MYB58/63 clade (Figure S3). Compared with rice, sorghum and *Brachypodium distachyon*, the expanded number of MYB58/63 members in switchgrass is due to its increased ploidy level. We named the genes *PvMYB58/63A*,* PvMYB58/63B*,* PvMYB58/63C and PvMYB58/63D*. Among them, *PvMYB58/63A* and *PvMYB58/63B* share 95% similarity at the amino acid level. All are highly co‐expressed with lignin biosynthesis genes (Dataset S1). Quantitative PCR analysis showed that *PvMYB58/63A* is more highly expressed in stem than in leaf blade and leaf sheaths, and is overall more highly expressed than *PvMYB58/63C* (Figure 2a). *In situ* hybridization was performed to investigate tissue‐specific expression. Because TFs are usually expressed at low levels, the signal‐to‐noise ratio was sometimes low in the *in situ* hybridization analyses presented in this paper, and data for results from replicate experiments are therefore shown (in main and Supplementary Figures). On the basis of *in situ* hybridization, *PvMYB58/63A* and *PvMYB58/63C* are expressed in multiple cell types in switchgrass stem cross sections (Figures 2b, S4 and S5). These results suggest that, like their homologs in Arabidopsis and rice, *PvMYB58/63s*, especially *PvMYB58/63A*, are probably involved in secondary wall formation, but that the switchgrass genes may also be associated with other processes.

To investigate the function of PvMYB58/63 *in vivo*, we generated overexpression‐ and down‐regulation‐ lines of PvMYB58/63A in transgenic switchgrass cultivar Alamo (Lowland ecotype). The RNA interference (RNAi) approach was used to achieve down‐regulation of the target gene. Transcript levels of the target gene in the transgenic lines were measured using qRT‐PCR (Figures 2c and S6). The selected overexpression and knockdown lines exhibited at least a 50‐fold increase and a 70% to 85% reduction in *PvMYB58/63* expression compared with that of the vector control plant respectively (Figures 2c and S6).

Compared with the controls, the PvMYB58/63A‐overexpressing plants displayed on average a 40% reduction in height (Figure 2d, e). Cross sections of mature stems were stained with phloroglucinol‐HCL to assess total lignin. All MYB58/63A‐overexpressors displayed increased staining in mature stem (Figure 2f). Increased total lignin content was confirmed by the AcBr method (1.1‐ to 1.3‐fold increase, Figure 2g) and by thioacidolysis (1.2‐ to 1.6‐ fold increase) in the whole tillers at the E4 stage (Figure 2h). In addition, an increased syringyl to guaiacyl (S:G) ratio was observed in the MYB58/63A overexpressors due to a disproportionate increase in S units (Figure 2i). qRT‐PCR analysis revealed, consistent with the co‐expression analysis, increased expression of *F5H* (Figure 2j) and all other lignin biosynthesis genes (except *C3′H*) in PvMYB58/63A‐overexpression lines (Figure S7). Besides, similar to a previous report on rice OsMYB58/63‐RNAi lines (Hirano *et al*., 2013b), no obvious effects of knockdown of *PvMYB58/63* were observed on plant growth, stem structure or lignin content, although several lignin biosynthesis genes showed reduced expression (Figure S8). This possibly reflects redundancy by homologous genes and/or other TFs. Taken together, our results show that PvMYB58/63A is an activator of secondary wall formation via up‐regulation of most lignin biosynthesis genes including F5H.

#### PvMYB42/85

**Figure 3:**
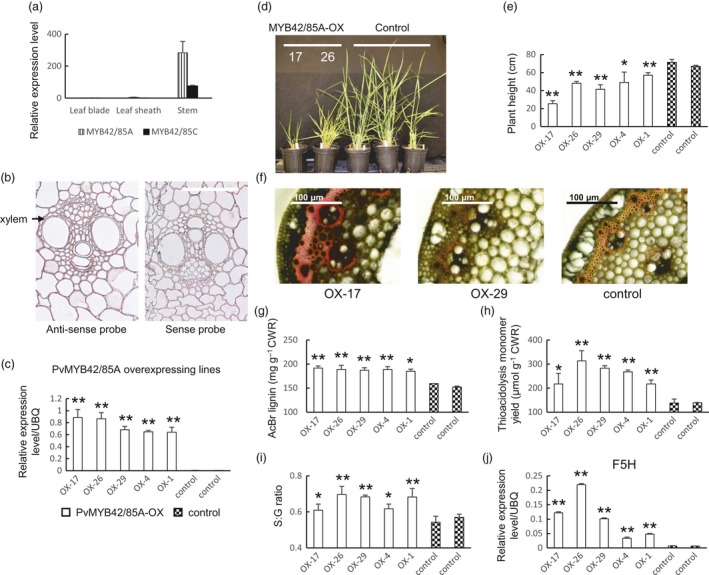
Phenotype of transgenic switchgrass overexpressing PvMYB42/85A. (a) qRT‐PCR analysis of PvMYB42/85A transcript levels in leaf blade, leaf sheath and stem. The transcript level in leaf blade was set to 1. (b) *In situ* hybridization of PvMYB42/85A in switchgrass stem, E4 stage, internode 2. Bars, 100 μm. (c) qRT‐PCR analysis of PvMYB42/85A transcripts in control and PvMYB42/85A‐OX transgenic switchgrass. UBQ, ubiquitin. (d) Representative photograph of control and PvMYB42/85A‐OX lines. (e) Plant height for control and PvMYB42/85A‐OX lines. (f) Phloroglucinol‐HCl staining of stem cross sections (internode 2 at the E2 stage) of PvMYB42/85A‐OX transgenic switchgrass. Bars, 100 μm. (g) Total lignin content of whole tillers at R1 stage determined by the AcBr method. (h) Total lignin content and (i) S/G ratio at R1 stage determined by thioacidolysis. (j) qRT‐PCR analysis of F5H transcripts in control and PvMYB42/85A‐OX transgenic switchgrass. All data are means ± SE (*n* = 3). Significant differences from the equivalent control were determined by the Student's *t*‐test and are represented by a single asterisk (*P* < 0.05) or double asterisk (*P* < 0.01).

Four genes were found to be homologs of *AtMYB42/85* in the switchgrass genome (Figure S3), and named *PvMYB42/85A, B, C* and *D*. Three of them were found to be co‐expressed with lignin biosynthesis genes. Among them, *PvMYB42/85A*, which shared 94% amino acid similarity with *PvMYB42/58B*, showed the highest expression in stems (Figure 3a). Although the signal with the sense probe was high, *in situ* hybridization suggested that both *PvMYB42/85A* and *PvMYB42/85C* were expressed throughout the stem, including vascular bundles and epidermal cells, with *PvMYB42/85A* being preferentially expressed in xylem vessels (Figures 3b, S4 and S9). Similar to PvMYB58/63**,** we generated overexpression and RNAi lines for PvMYB42/85A in transgenic switchgrass. PvMYB42/85A transcript levels exhibited at least a 50‐fold increase in selected PvMYB42/85‐overexpressing lines, and at least 70% reduction in RNAi lines based on qPCR (Figures 3c and S6). A similar phenotype was observed in the PvMYB42/85A overexpressors as in the PvMYB58/63A overexpressors. This comprised reduced plant height (36% reduction on average, Figures 3d, e and S10), increased phloroglucinol staining of stem cross sections (Figures 3f and S11), elevated total lignin content (1.2‐fold on average by the AcBr method, Figure 3g; 1.8‐fold on average by the thioacidolysis method, Figure 3h), increased S:G ratio (Figure 3i) and increased transcript levels of F5H and the other lignin biosynthesis genes (Figures 3j and S12). No comparable opposite changes were detected in the PvMYB42/85‐RNAi line although several lignin biosynthesis genes were down‐regulated (Figure S13). PvMYB42/85A, like PvMYB58/63A, therefore activates lignin biosynthesis via expression of lignin biosynthesis genes including F5H.

#### PvWRKY12

**Figure 4:**
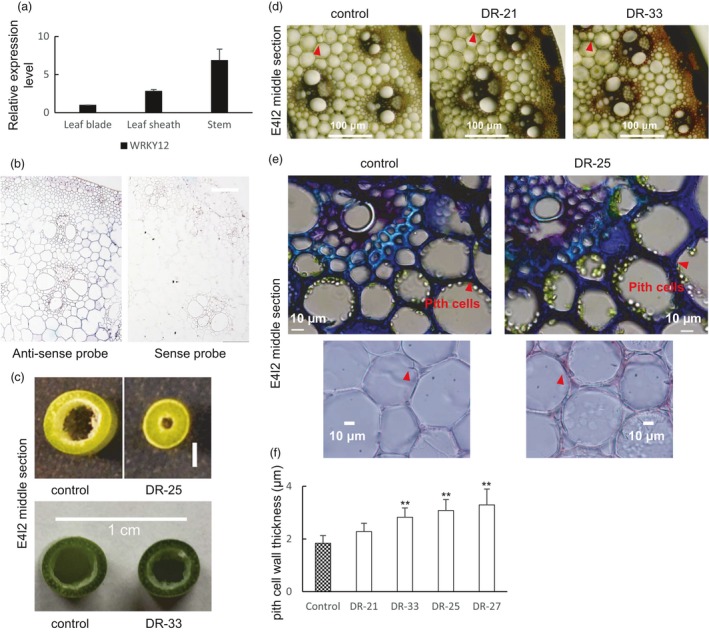
Phenotype of transgenic switchgrass with PvWRKY12 down‐regulated by the dominant repressor strategy (Gallego‐Giraldo *et al*., 2016). (a) qRT‐PCR analysis of PvWRKY12 transcript levels in leaf blade, leaf sheath and stem. The transcript level in leaf blade was set to 1. (b) *In situ* hybridization of PvWRKY12 in switchgrass stem, E4 stage, internode 2. Bars, 100 μm. (c) Representative photograph of stem sections from control and PvWRKY12‐DR lines. (d) Phloroglucinol‐HCl staining of stem cross sections of PvWRKY12‐DR transgenic switchgrass, internode 2 at the E4 stage. The arrows indicate pith cell wall. Bars, 100 μm. (e) Stem cross sections of PvWRKY12‐DR transgenic switchgrass. The arrows indicate pith cell wall. Bars, 10 μm. (f) Measurement of pith cell wall thickness in stems of PvWRKY12‐DR transgenic switchgrass. E4‐2, internode 2 of E4 tillers for (c–f). All data are means ± SE (*n* = 3). Significant differences from the equivalent control were determined by the Student's *t*‐test and are represented by a single asterisk (*P* < 0.05) or double asterisk (*P* < 0.01).

Neither *AtWRKY12* nor its homolog *PvWRKY12* appeared in the co‐expression network of lignin biosynthesis genes in Arabidopsis and switchgrass. This is consistent with WRKY12 not being a direct regulator of lignin formation (Wang *et al*., 2010). Quantitative PCR analysis revealed that *PvWRKY12* is expressed in leaf blades, leaf sheaths and stems, with the highest transcript abundance in stems (Figure 4a), and *in situ* hybridization of mature stem tissue sections suggested highest expression in pith parenchyma cells (Figures 4b and S14), consistent with the proposed role of this gene as a repressor of lignification in this cell type (Wang *et al*., 2010).

We have previously reported the generation of WRKY12‐down‐regulated transgenic lines of switchgrass using the dominant repressor (DR) strategy (Gallego‐Giraldo *et al*., 2016). These showed a severe to mild phenotype of reduced plant growth and disruption of xylem vessels and tracheids in the stems (Gallego‐Giraldo *et al*., 2016). We here further extend this analysis. Compared with the controls, the relative thickness of the stems relative to the central cavity was increased in PvWRKY12‐DR lines, but with decreased overall diameter of the stem (Figures 4c and S15). Phloroglucinol staining of cross sections of mature stems showed an enhanced lignification in epidermal cells and vascular bundles in the PvWRK12‐DR lines (Figure 4d). Furthermore, the thickness of the pith cell walls was significantly increased in PvWRKY12‐DR lines (Figure 4d–f), consistent with previous reports of Arabidopsis and Medicago *wrky12* mutant lines utilizing the same approach (Wang *et al*., 2010).

#### PvSWN2 and PvSND2

*PvSWN2A* (AP13ITG56117RC_at) and *PvSWN2B* (KanlowCTG11938_s_at), close homologs of the Arabidopsis secondary cell wall master regulator *SND1*/*NST1*/*2*, showed co‐expression with six and eight lignin biosynthesis genes, respectively, in switchgrass (Dataset S1). We generated more than 10 independent PvSWN2‐RNAi lines in switchgrass of which six exhibited strongly reduced expression of the target genes (Figure S16). Compared with the vector control, no obvious changes in plant growth, lignin content or stem structure were observed in any of the PvSWN2‐RNAi lines, except slightly increased S:G ratio in two lines (Figure S16). Quantitative PCR analysis showed that the decreased expression of *PvSWN2* in the transgenic lines resulted in no obvious effects on the expression of either secondary cell wall‐related transcription factors (PvSWN1, PvMYB4, PvMYB46, PvMYB58/63 and PvMYN42/85) or F5H (Figure S16). This suggests that PvSWN2 may be functionally redundant with other TFs (e.g. PvSWN1) and not exclusively regulate the expression of *F5H*.

The expression of genes grouped in the SND2 clades (Figure S17) shows correlation with multiple lignin biosynthesis genes in both Arabidopsis (AT4G28500) and switchgrass (KanlowCTG43583_s_at, AP13CTG15049_s_at, AP13ITG71892_at, AP13ITG74807_s_at and KanlowCTG37619_s_at) (Dataset S1). To explore the potential role of PvSND2 in switchgrass secondary wall formation, more than 10 PvSND2 RNAi lines were generated and a selection confirmed by qPCR analysis (Figure S18A). Reduced expression of *MYB103 and CESA4* and *CESA9* (two cellulose biosynthesis genes involved in secondary wall formation) was detected in the transgenic lines compared with the vector control. Cell wall polysaccharide analysis indicated a reduced content of hemicellulose in one transgenic line (Figure S18B). In addition, the relative amount of galactose and glucose in the hemicellulose fraction decreased in all transgenic lines and in one transgenic line respectively (Figure S18C). No significant changes were observed in plant growth, lignin content or stem structure (Figure S19). The results suggest that PvSND2 may be involved in secondary wall‐related cellulose and hemi‐cellulose biosynthesis.

### An extended co‐expression analysis for selected TFs

To further evaluate the potential role of TFs in secondary wall formation and other biological pathways in switchgrass, we extended the co‐expression network by adding 19 and 21 TFs as bait genes in Arabidopsis and switchgrass respectively. These TFs included WRKY12, SWNs, MYB46/83, MYB4, MYB58/63 and MYB42/85 (Table S3). A hierarchical structure of correlated‐relationships among the genes was observed in both the Arabidopsis and switchgrass networks (Dataset S2).

**Figure 5:**
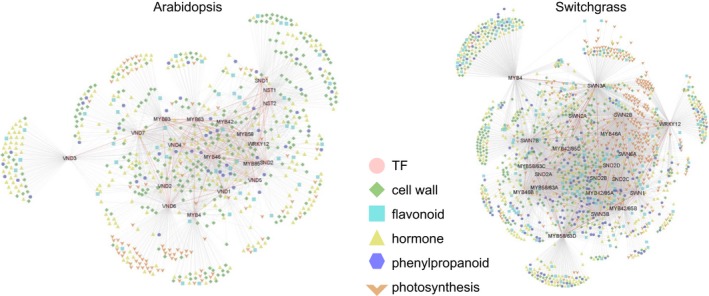
Extended co‐expression network of Arabidopsis and switchgrass using selected transcription factors as baits. Genes involved in selected biological functions (cell wall, flavonoid, hormone, phenylpropanoid and photosynthesis) were present in the network. Red lines represent the correlated relationship between bait genes. The Arabidopsis SWNs were named as VND (orthlog of switchgrass PvSWN3‐7) and NST (orthlog of switchgrass PvSWN1 and PvSWN2) (Zhong *et al*., 2010, 2015).

There were clear similarities in the association between TFs in Arabidopsis and switchgrass (Figure 5). The coordinated expression of *AtWRKY12* with *AtNST1*,* AtNST2* and *AtSND1* is consistent with the observation that AtWRKY12 can directly regulate the expression of downstream NAC master switches (Wang *et al*., 2010, 2011). Similarly, *PvWRKY12* shows co‐expression with *PvSWN1* and *PvSWN2B* (the homologs of *AtNST1*,* AtNST2* and *AtSND1*). *MYB46* is tightly co‐expressed with *SWN* and *MYB42/85* and *MYB58/63* in both Arabidopsis and switchgrass. This suggests a central role of MYB46, controlled by upstream SWNs and modulating downstream TFs in the transcriptional regulatory programme. In contrast, MYB4, the secondary wall repressor, is less co‐expressed with other TFs in switchgrass compared with its homolog in Arabidopsis. We suggest that expression of *MYB4* in switchgrass is partially independent of the NAC‐MYB46‐MYB transcriptional regulatory cascade.

To understand the functional differentiation of genes co‐expressed with the above TFs in Arabidopsis and switchgrass, we inferred the biological pathways in which these genes are significantly enriched, using the Fisher exact test (Figure S20). Consistent with the involvement of the TFs in secondary wall formation, many co‐expressed genes in Arabidopsis and switchgrass are enriched in the functional pathways of cell wall degradation, cell wall modification, cellulose/hemicellulose synthesis and phenylpropanoid synthesis. Besides cell wall‐related genes, groups of genes assigned to flavonoid biosynthesis, hormone and photosynthesis pathways were also tightly co‐expressed with these TFs in both species (Figure 5). The appearance of conserved co‐expression modules in the Arabidopsis and switchgrass co‐expression networks suggests that these TFs may be involved in the coordination and crosstalk between cell wall development and other biological pathways in both species.

### RNA‐seq analysis of selected TF transgenic lines

To investigate the global changes in gene expression modulated by secondary wall TFs in switchgrass, we performed RNA sequencing on four of the previously generated transgenic switchgrass lines (MYB4‐OX, MYB58/63A‐OX, MYB42/85A‐OX and WRKY12‐DR). For each set of transgenic plants, we selected one strongly expressing line, one or two weakly expressing lines, and one control line (Table S4), and generated transcriptomes for internode tissues and whole tillers (Datasets S3 and S4). Pearson correlation and cluster (PCC) analysis of transcript profiles were used to assess the level of correlation between biological replicates (Figure S21). Overall, PCC values ranged from 0.70 to 0.99 within replicates.

**Figure 6:**
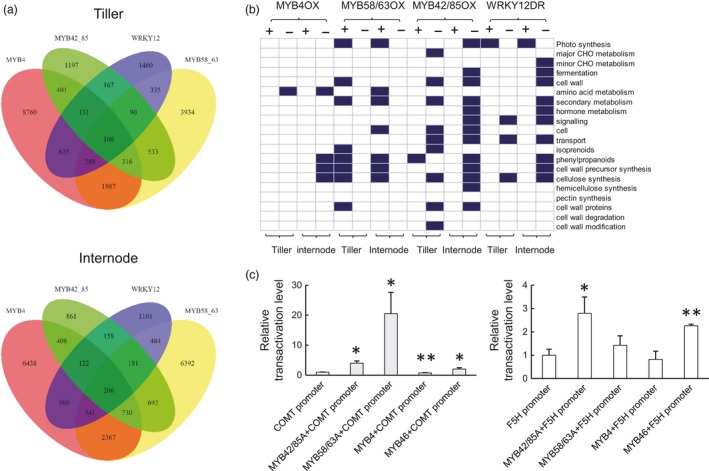
Overview of RNA‐seq analysis of switchgrass lines with modified expression of TFs, and ability of TFs to activate lignin gene promoters. (a) Venn diagrams showed the number of genes differentially expressed in tillers and internodes of PvMYB4OX, PvMYB58/63OX, PvMYB42/85OX and PvWRKY12DR transgenic lines compared with control. (b) Functional distribution of genes differentially expressed in tillers and internodes of PvMYB4OX, PvMYB58/63OX, PvMYB42/85OX and PvWRKY12DR transgenic lines. Blue represents functional groups that were significantly enriched (determined by Fisher exact test, FDR<0.1). (c) Trans‐activation of the PvCOMT and PvF5H promoters by PvMYB4, PvMYB46, PvMYB58/63A and PvMYB42/85A. Firefly luciferase activities were quantified and normalized to Renilla luciferase activities. The activities in the protoplasts transfected with promoter‐reporter construct and an empty effector construct were set to 1. All data are means ± SE (*n* = 3). Significant differences for the activation of PvCOMT and PvF5H promoters were determined by the Student's *t*‐test and are represented by a single asterisk (*P* < 0.05) or double asterisk (*P* < 0.01).

Thousands of genes were identified to be differentially expressed in the TF strongly expressing lines compared with the controls (Figure 6a and Dataset S5). The MYB4‐OX and MYB58/63A‐OX lines displayed the highest abundance of differentially expressed genes in tillers (11,112 genes in MYB4‐OX and 7,484 genes in MYB58/63A‐OX) and internodes (12,509 genes in MYB4‐OX and 11,393 genes in MYB58/63A‐OX) (Figure 6a and Table S5), consistent with their severe phenotypes compared with the control. Accompanying the opposing lignification phenotypes between MYB4‐OX and MYB58/63A‐OX, 900 and 1,162 genes, respectively, exhibited opposite expression patterns in tillers and internodes between the two transgenic lines (Dataset S3 and S4).

The enrichment analysis of differentially expressed genes on biological pathways showed that the significantly enriched functional categories included phenylpropanoid, cellulose and cell wall precursor synthesis (Figure 6b). A further survey of the differentially expressed genes grouped in the cell wall‐related categories indicated that the four TFs display both commonalities and differences in target genes, and differential levels of regulation on target genes in internode and stem (Table S6). PvMYB4 and PvMYB58/63A altered the expression of all lignin biosynthesis genes (except *C3′H*) in opposite directions in both internodes and tillers, whereas PvMYB42/85A significantly activated lignin biosynthesis genes with the exception of *PAL*,* C3′H* and *CCoAOMT* in tillers. Furthermore, the weakly overexpressing PvMYB4 and PvMYB58/63 lines exhibited altered expression of *4CL* and *HCT*, and *CCR* and *F5H* respectively (Table S5). Thus, PvMYB4, PvMYB58/63A and PvMYB42/85 can be considered as regulators of lignin biosynthesis genes with different preferential targets.

An overall decrease of gene expression involved in flavonoid and both primary and secondary cell wall cellulose biosynthesis was detected in PvMYB4‐OX lines in internodes, but this effect was much weaker or did not occur at all in tillers (Table S6). In contrast, PvMYB58/63A overexpression strongly up‐regulated genes involved in flavonoid and secondary cell wall cellulose biosynthesis in both internodes and tillers (Table S6).

We next assessed the effects of the four TFs on expression of other TFs (Tables S5 and S6). The RNAseq data confirmed that expression of the *MYB4*,* MYB58/63*,* MYB42/85* and *WRKY12* target genes was strongly altered in the corresponding transgenic lines, consistent with qPCR analysis. The transcript level of *SWN1, SWN2, PvMYB46, MYB42/85, MYB32, MYB103* and *SND2* was significantly increased in internodes and tillers of the PvMYB58/63‐OX line, whereas the expression of these genes was decreased in internodes but not (or less) affected in tillers of the PvMYB4‐OX and PvMYB42/85‐OX lines. This suggests an opposite direction of regulation by MYB58/63, and MYB4 and MYB42/85, in the internode, but not in the tillers. Furthermore, *MYB58/63*,* MYB4* and *MYB42/85* are considered as downstream targets of SWN1, SWN2 and MYB46 (McCarthy *et al*., 2009; Zhong *et al*., 2007, 2011, 2015). Our data suggest a ‘feed‐back’ regulation in the NAC‐MYB‐based transcriptional network of secondary wall development in switchgrass. Interestingly, no significant changes in expression of SWNs were observed in the WRKY12‐DR lines compared with the control (Tables S5 and S6), a result that was further confirmed by q‐PCR (Figure S22).

To explore the potential target genes of PvWRKY12, we combined the RNA‐seq and co‐expression analysis to discover 125 genes that were differentially expressed in both internodes and tillers of PvWRKY12‐DR lines and showed correlated expression with PvWRKY12 in large scale‐ microarray data sets (Dataset S6). Among them, we found an increased expression of a homolog of the bZIP63 transcription factor (Pavir.Cb00258) and decreased expression of a homolog of the bZIP44 transcription factor (Pavir.Fb01406) and two WRKY41 homologs (Pavir.Bb02088 and Pavir.J16737) in the PvWRKY12DR line. Arabidopsis bZIP63, bZIP44 and WRKY41 are involved in glucose signalling, cell wall modification and multiple developmental processes respectively (Duan *et al*., 2018; Iglesias‐Fernandez *et al*., 2013; Matiolli *et al*., 2011). We suggest these genes could be considered as candidate targets of PvWRKY12.

### Promoter transactivation assays

To directly confirm the transcriptional regulation of lignin biosynthesis genes by TFs, we performed transient promoter transactivation analysis using a promoter‐luciferase reporter system. The reporter vectors, which contained the promoters of switchgrass *F5H* and *COMT* (two major genes in lignin biosynthesis) driving the firefly luciferase gene, were co‐transfected into Arabidopsis protoplasts with four separate TF effectors (MYB4, MYB58/63A, MYB42/85A and MYB46) under the control of the constitutive cauliflower mosaic virus 35S promoter (Figures 6c and S23). Expression from the *PvCOMT* promoter was significantly activated by PvMYB58/63A, PvMYB42/85A and PvMYB46, and weakly down‐regulated by PvMYB4. In contrast, the *PvF5H* promoter was significantly but only weakly activated by PvMYB42/85A and PvMYB46, but was not activated by PvMYB58/63A or repressed by PvMYB4 (Figures 6c and S23). These results directly confirm the ability of PvMYB4, PvMYB58/63 and PvMYB42/85 to regulate the expression of lignin biosynthesis genes, but the observed in planta modulation of *F5H* expression on overexpressing PvMYB4 and PvMYB58/63A may not be through direct recognition of its promoter region by these TFs, or may require additional co‐acting TFs.

## Discussion

### An improved strategy to identify regulators and their target candidates using co‐expression analysis

Co‐expression analysis reflects the correlated expression of pairs of genes. It is a powerful tool to decipher transcriptional regulatory relationships because the expression of TFs tends to be transcriptionally coordinated with that of their functional targets (Hansen *et al*., 2014). The first step in co‐expression network construction is to measure the correlation of expression values for genes in a pairwise fashion (Serin *et al*., 2016). Many methods have been designed to determine the degree of correlation between gene pairs under global conditions or given conditions (Serin *et al*., 2016). However, strongly correlated expression of genes may occur under some ‘to‐be‐identified’ conditions (Li *et al*., 2009). Here, we developed a method based on a bi‐clustering algorithm (Li *et al*., 2009; Zhang *et al*., 2017) to assess the strength of the correlated relationships. This method is effective and efficient in detecting correlated relationships between pairs of variables under both all and partial conditions (see Methods S1).

Using this method, we constructed large scale co‐expression networks of switchgrass and Arabidopsis using lignin biosynthesis genes as bait genes. The networks contained 645 and 1274 TFs in Arabidopsis and switchgrass, respectively, which could be associated with secondary wall development. The distribution of TF families was consistent with a previous survey of co‐expressed TFs in secondary wall formation in Arabidopsis, rice, maize and sugarcane identified by Pearson correlation, Spearman correlation and mutual rank methods (Cassan‐Wang *et al*., 2013; Ferreira *et al*., 2016; Hansen *et al*., 2014; Hirano *et al*., 2013a; Ruprecht *et al*., 2011), supporting the validity of our co‐expression method to identify co‐expression modules in large‐scale data sets. Of these conserved TF families, multiple TF subclades such as SWNs, SNDs, MYB46, MYB4/32, MYB42/85 and MYB58/63 have been identified previously as playing a role in secondary cell wall regulation in Arabidopsis (Nakano *et al*., 2015; Zhong and Ye, 2015; Zhong *et al*., 2010). The experimental validation of these TFs using transgenic technology in the present work confirmed their role in switchgrass secondary wall development. To explore the potential targets of the TFs, we extended the co‐expression networks by adding 21 TFs as bait genes and conducted transcriptome sequencing on TF transgenic lines. We found that many genes were co‐expressed with certain TFs that appeared to be differentially expressed in the corresponding TF transgenic line. Comparative co‐expression analysis across plant species is a feasible strategy for initial investigation of transcriptional regulators using structural genes, and for target genes using transcriptional regulators.

### Commonalities and differences in transcriptional regulation of secondary wall formation in switchgrass and Arabidopsis

Grasses and dicots, divergent after the establishment of vascular plants, may share conserved functionalities in the transcriptional networks that regulate their secondary wall formation (Rao and Dixon, 2018; Zhong *et al*., 2010). The appearance of orthologous TFs in co‐expression networks of lignin biosynthesis, and the similarity of network structure in the TF‐extending co‐expression networks in Arabidopsis and switchgrass suggest that these TFs may play similar roles in secondary wall formation in both species. Here, we investigated the roles of MYB4, MYB58/63, MYB42/85, WRKY12, SWN2 and SND2 in the secondary cell wall biosynthesis programme in switchgrass. The phenotypes of the transgenic lines with altered expression of these TFs displayed many similarities to those of the corresponding lines in Arabidopsis. For example, overexpressing either *AtMYB58/63* in Arabidopsis (Zhou *et al*., 2009) or *PvMYB58/63* in switchgrass caused reduced plant height, increased lignin content, and elevated expression of monolignol biosynthesis, and down‐regulating *PvWRKY12* in switchgrass resulted in enhancement of pith cell walls, similar to the phenotype of the *atwrky12* mutant (Wang *et al*., 2010). However, we also detected several differences in secondary wall development between switchgrass and Arabidopsis.

Although both PvMYB58/63 and AtMYB58/63 function as activators of lignin biosynthesis, an increased expression of secondary wall‐associated cellulose synthase and xylan synthase genes, as well as flavonoid biosynthesis genes, was observed here in PvMYB58/63‐OX plants but not in Arabidopsis MYB58/63 overexpressors (Zhou *et al*., 2009). Considering that OsMYB58/63 could also up‐regulate secondary wall‐related cellulose synthase genes in transactivation assay (Noda *et al*., 2015), and the overexpression of *SbMYB60* (the ortholog of *AtMYB58/63*) in sorghum altered cell wall cellulose and xylan content (Scully *et al*., 2016), we suggest that the MYB58/63 clade in grasses may function as a broader activator regulating both lignin and cellulose/hemicellulose biosynthesis, rather than possessing the ‘lignin‐ specific regulator’ function ascribed to AtMYB58/63 in Arabidopsis.

Differences in regulation of target lignin biosynthesis genes were also observed between orthologous Arabidopsis and switchgrass TF genes. Differences in co‐expression modules suggested that Arabidopsis and switchgrass may operate differently to regulate F5H, the entry point to S lignin biosynthesis; Arabidopsis may assign an additional group of TFs to regulate the expression of *F5H*, whereas switchgrass may utilize the common TFs that regulate the expression of other lignin biosynthesis genes. In Arabidopsis, the transcript abundance of F5H is influenced by SND1 and MYB103 (Ohman *et al*., 2013; Zhao *et al*., 2010). In contrast, while decreased expression of *F5H* was not observed in PvSWN2‐DR lines, we found that PvMYB42/85 and PvMYB46 directly activated the expression of *PvF5H*, and significantly altered expression of *PvF5H* occurred in PvMYB4 and PvMYB58/63 overexpressor lines. All four MYB TFs had the capability to modulate other lignin biosynthesis genes, which may contribute to the correlated expression of *F5H* with other lignin biosynthesis genes in switchgrass.

**Figure 7:**
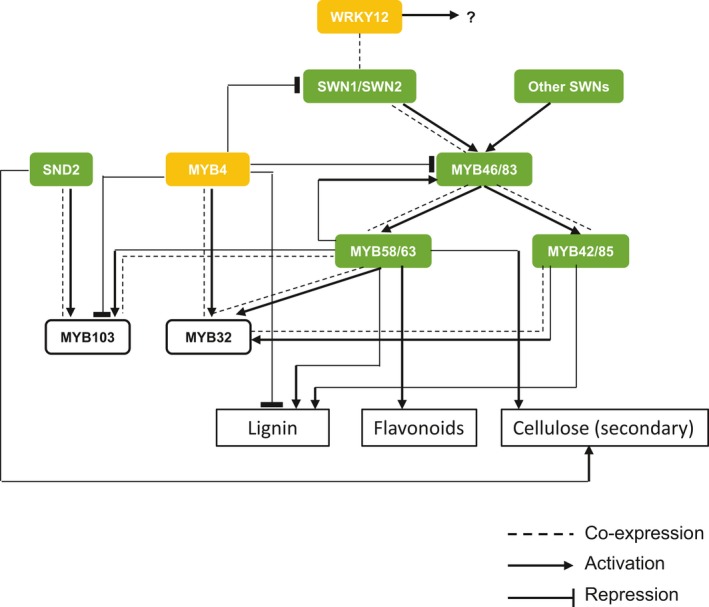
Proposed transcriptional network for secondary cell wall formation in switchgrass. Yellow and green colours represent negative and positive regulators respectively. Arrows and bars at the ends of lines represent positive and negative transcriptional regulation respectively. Dashed line indicates co‐expression relationship.

In conclusion, switchgrass displays a complex transcriptional network for regulation of its secondary wall biosynthetic programme. This shares many common features with the secondary wall regulatory programme in Arabidopsis, but also exhibits several differences. The network, supported by transgenesis, RNA‐seq analysis, transactivation and co‐expression analyses, is summarized in Figure 7. Our analyses identify gene targets for the potential modification of multiple cell wall components in switchgrass.

## Experimental procedures

### Co‐expression analysis

For co‐expression analysis, the Arabidopsis microarray database was downloaded from NCBI GEO website (GSE34188). The switchgrass microarray database was accessed from the Noble Foundation Switchgrass Functional Genomics Server at http://switchgrassgenomics.noble.org/ (Zhang *et al*., 2013). The RMA method was applied to normalize these Affymetrix microarray data. A new approach based on our previous bi‐clustering method (Li *et al*., 2009; Zhang *et al*., 2017) was used to detect the co‐expression modules in Arabidopsis and switchgrass. The details of this co‐expression method, and comparison to the standard Pearson correlation analysis, are provided in Methods S1 and Dataset S7. The co‐expression network was visualized using the program Cytoscape (Shannon *et al*., 2003).

### Plant materials, transformation and growth conditions

Switchgrass cultivar Alamo was grown in the greenhouse under 28 °C with 16 h light. For plant transformation, the pANIC vector (Mann *et al*., 2012) was used for plasmid construction. Briefly, for overexpression constructs, the full length of coding sequence was cloned into pANIC vector under the control of the *ZmUbi1* promoter (Mann *et al*., 2012); for RNAi constructs, the partial coding sequences were cloned into pANIC‐RNAi binary vector under the control of the *ZmUbi1* promoter (Mann *et al*., 2012); for dominant repressor constructs, the PvWRKY12‐DR sequence was cloned and inserted into the destination vector pANIC10A as described (Gallego‐Giraldo *et al*., 2016). All primers used for cloning are listed in Table S7. The switchgrass NFCX01 line was used for stable transformation. Agrobacterium‐mediated transformation in switchgrass was performed as described previously (Shen *et al*., 2012; Xi *et al*., 2009).

### Quantitative reverse transcription

RNA isolation, cDNA synthesis, and real‐time RT‐PCR were performed as previously described (Rao *et al*., 2016). For determination of tissue‐specific expression, total RNA was isolated from leaf, leaf sheath and stem of switchgrass at the reproduction (R1) developmental stage (Hardin *et al*., 2013). qRT‐PCR was performed with three biological replicates for each gene tested. Data were collected using an optical 384 well plate with QuantStudio™ 6 Flex (Applied Biosystems, Foster City, CA). The *Ubi* gene was used as a reference for relative quantification of transcript levels. All primers designed for qRT‐PCR are listed in Table S7.

### *In situ* hybridization

Samples of second internodes of at least ten switchgrass plants at the E4 stage (the last stage of tiller elongation before appearance of floral organs) (Hardin *et al*., 2013; Shen *et al*., 2009) were harvested for *in situ* hybridization. This stage, immediately prior to R1, was used because the additional lignification at the R1 stage made the tissue hard to section. The tissue preparation including fixation, dehydration and paraffin embedding was performed according to Long's protocol (http://www.its.caltech.edu/~plantlab/protocols/insitu.pdf). Pre‐hybridization, hybridization, washing and imaging were performed as described previously (Rao *et al*., 2016).

### Determination of lignin content

Switchgrass whole tillers were collected at the R1 stage to prepare cell wall residues (CWR) by sequential extraction with chloroform/methanol (1:1), 100% methanol, 50% methanol and water (three times each). Twenty‐five milligrams of CWR was used for lignin analysis. The acetyl bromide method and thioacidolysis method followed by GC‐MS were used to determine total lignin content and quantify lignin‐derived monomers respectively. All analytical methods were performed as previously described (Shen *et al*., 2009).

### Cell wall polysaccharide analysis

Switchgrass whole tillers were ground in liquid nitrogen, and washed in organic solvent to prepare the alcohol insoluble residue (AIR) as described previously (Willis *et al*., 2016). Cell walls were obtained after removing starch from the AIR using Novazyme specific enzymes. Cell walls were treated with 2N trifluoroacetic acid (TFA) to solubilize and hydrolyze non‐cellulosic polysaccharides. The amount of sugar solubilized was determined using the phenol‐sulphuric acid colorimetric assay (DuBois *et al*., 1956). The identity of the solubilized sugars was determined by GC‐FID analysis after derivatization of the sugars to their corresponding alditol acetates (York *et al*., 1986). The insoluble material after the TFA hydrolysis was washed extensively and the pellets were used to determine the cellulose content. The cellulose was hydrolyzed using the Shaeman hydrolysis method as described previously (Selvendran *et al*., 1979) and the amount of glucose solubilized was determined by phenol‐sulphuric acid colorimetric assay.

### Microscopic analysis

Switchgrass internode samples for lignin staining were collected and cut with a vibratome (Thermo Fisher Scientific, Germany). The second internode at the E4 stage was used for lignin staining in the case of the PvWRKY12‐DR lines. However, stems at the E4 stage of MYB58/63‐OX lines and MYB42/85‐OX lines were difficult to section due to high lignin deposition. Instead, cross sections were cut from the top part of the second internode at the E2 stage for lignin staining in these lines. Vascular tissues, parenchyma cells and interfascicular fibers have been shown to be significantly lignified in switchgrass at this stage (Shen *et al*., 2009; Sarath *et al*., 2007). Phloroglucinol‐HCL staining was carried out as previously described (Shen *et al*., 2009).

For determination of cell wall thickness, E4I2 stage samples were longitudinally sectioned to 60 μm with a Leica CM 1850 cryostat and observed with a Nikon microphot‐FX microscope. The process of fixation/cryoprotection was as previously described (Wang *et al*., 2010), and the pith cell wall thickness was measured based on a scale bar at the μm level (Wang *et al*., 2010). Three cell walls were measured in each of three separate sections.

### Next generation sequencing and RNA‐seq analysis

The whole tillers at R1 stage (Hardin *et al*., 2013; Shen *et al*., 2009) were harvested as ‘tiller’ samples and middle sections from internode 4 stems were cut from the tillers as ‘internode’ samples. All samples were taken at the same time of day (between 1 and 3 pm) and stored at −80 °C for RNA extraction. Each sample was submitted for 150 bp paired‐end sequencing to generate 40–50 million reads. Each sample group had at least two biological replicates. Paired‐end Illumina reads after filtering and trimming treatment by in‐house perl scripts (Rao *et al*., 2016) were mapped to the Switchgrass genome *Panicum virgatum* v1.1 (http://phytozome.jgi.doe.gov/) using Bowtie 2 (v2.3.2.0) (Langmead and Salzberg, 2012) and TopHat (v2.2.1) (Kim *et al*., 2013) with default parameters. Differentially expressed genes between the controls and transgenic lines were determined using Cufflinks (v2.2.1) (Trapnell *et al*., 2013) with default setting of adjusted *P*‐value < 0.05. As previously described (Rao *et al*., 2016), classification for differentially expressed genes was based on MapMan mappings of their Arabidopsis homologs (Thimm *et al*., 2004). Significant functional enrichment was inferred using Fisher's exact test with Benjamini–Hochberg multiple testing correction [false discovery rate (FDR) ≤ 0.1].

### Transactivation assays

The effector constructs were generated by inserting coding sequences of MYB TFs after the 35S promoter of the Gateway overexpression vector P2GW7 (Karimi *et al*., 2002). The reporter constructs were generated by inserting promoters of lignin biosynthesis genes into the vector P2GWL7 (Wang *et al*., 2010). Primers used for plasmid construction are listed in Table S7. The effector and reporter plasmids were co‐transfected into Arabidopsis leaf protoplasts isolated according to a previous report (Wang *et al*., 2010). Promoter activities are presented as Firefly LUC/Renilla LUC activities, and normalized to the value obtained from protoplasts transformed with empty effector vector. The data were the average of three biological replicates.

### Statistical analysis

Experimental data were subjected to statistical treatment by the Student's *t*‐test (Microsoft office Excel 2013). Significant difference between two groups was determined by *P*‐value < 0.05 and indicated by asterisks above bars.

### Accession numbers

The gene identification numbers in the switchgrass genome (*Panicum virgatum* 1.1) used in this study are PvMYB58/63A (Pavir.Gb00587), PvMYB58/63B (Pavir.Ga00752), PvMYB58/63C (Pavir.Aa01159), PvMYB58/63D (Pavir.J10932), PvMYB42/85A (Pavir.Bb02654), PvMYB42/85B (Pavir.Ba01239), PvMYB42/85C (Pavir.Fb02321), PvMYB42/85D (Pavir.J36915), PvMYB4 (Pavir.J16675), PvWRKY12 (Pavir.Ga00648), PvSND2A (Pavir.Eb02718), PvSND2B (Pavir.J05241), PvSND2C (Pavir.Ca00926) and PvSND2D (Pavir.J04559). PvSWN1 (Pv.J07835), PvSWN2A (Pv.J20698), PvSWN2B (Pv.J21162), PvMYB46A (Pavir.J11191) and PvMYB46B (Pavir.Ca02370) were previously described in Zhong *et al*., 2015.

The data sets supporting the results of this article are available in the NCBI Sequence Read Archive (SRA) repository, NCBI SRA accession no. PRJNA480162.

## Conflict of interest

The authors declare they have no conflict of interest.

## Authors’ contributions

X.R and R.A.D designed the experiments; X.R., X.C., Q.M., Y.T. and W.Y., S.L. and F.C. collected and analysed the data; H.S., G.L., M.P., T.F. and X.X performed the experiments; X.R. and R.A.D. wrote the manuscript; R.A.D. obtained funding and is responsible for this article. All authors read and approved the manuscript.

## Supporting information


**Figure S1** Phylogenetic trees of MYB, NAC, bHLH, ERF and WRKY TFs that appear in Arabidopsis and switchgrass co‐expression networks of lignin biosynthesis.
**Figure S2** Co‐expression network of MYB and NAC TFs with lignin biosynthesis genes in Arabidopsis and switchgrass.
**Figure S3** Phylogenetic tree of MYB58/63 and MYB42/85 orthologs from Arabidopsis, Medicago, poplar, switchgrass, maize, rice, sorghum and *Brachypodium*.
**Figure S4** *In situ* hybridization of PvMYB58/63C and PvMYB42/85C in switchgrass stem.
**Figure S5** Additional *in situ* hybridization images for MYB58/63A.
**Figure S6** qRT‐PCR analysis of target transcripts in leaf of PvMYB58/63‐RNAi and PvMYB42/85‐RNAi transgenic switchgrass.
**Figure S7** qRT‐PCR analysis of lignin biosynthesis genes in leaf of PvMYB58/63A‐OX transgenic switchgrass.
**Figure S8** Phenotype of PvMYB58/63‐RNAi transgenic lines.
**Figure S9** Additional *in situ* hybridization images for MYB42/85A.
**Figure S10** Phenotypes of additional PvMYB42/85‐OX29, OX4 and OX1 plants in comparison to control lines.
**Figure S11** Lignin staining for PvMYB42/85 lines.
**Figure S12** qRT‐PCR analysis of lignin biosynthesis genes in leaf of PvMYB42/85A‐OX transgenic switchgrass.
**Figure S13** Phenotype of PvMYB42/85‐RNAi transgenic lines.
**Figure S14** Additional *in situ* hybridization images for WRKY 12.
**Figure S15** Stem outer diameters and stem radial thickness in PvWRKY12‐DR transgenic lines.
**Figure S16.** Phenotype of PvSWN2‐RNAi transgenic lines.
**Figure S17** Phylogenetic tree of NST and SND2 orthologs from Arabidopsis, poplar, maize, rice and switchgrass.
**Figure S18** Cell wall‐related gene expression and cell wall component analysis in PvSND2‐RNAi transgenic switchgrass.
**Figure S19** Phenotype of PvSND2‐RNAi transgenic lines.
**Figure S20** Functional distribution of genes co‐expressed with TFs in Arabidopsis and switchgrass.
**Figure S21** Correlation matrix of switchgrass transcriptomes determined by the Pairwise Pearson correlation coefficients (PCC) method.
**Figure S22** qRT‐PCR analysis of SWN1 and SWN2 in PvWRKY12DR transgenic switchgrass.
**Figure S23.** Trans‐activation assays of the PvCOMT and PvF5H promoters by PvMYB4.
**Table S1** Lignin biosynthesis genes as bait genes for co‐expression analysis in Arabidopsis and switchgrass
**Table S2** Numbers of TFs of different families that are co‐expressed with lignin biosynthesis genes in Arabidopsis and switchgrass.
**Table S3** Secondary wall‐related TFs as bait genes for co‐expression analysis in Arabidopsis and switchgrass.
**Table S4** Numbers of differentially expressed genes in RNA‐seq analysis of PvMYB4OX, PvMYB58/63OX, PvMYB42/85OX and PvWRKY12DR switchgrass lines.
**Table S5** Expression of cell wall‐related genes in all switchgrass transgenic lines.
**Table S6** Differential expression of secondary cell wall‐related genes in tillers and internodes of PvMYB4OX, PvMYB58/63OX, PvMYB42/85OX and PvWRKY12DR transgenic switchgrass lines compared with control.
**Table S7** Sequences for the gene‐specific primers used in this work.
**Methods S1** The method for co‐expression analysis.

**Dataset S1** Co‐expression analysis of lignin biosynthesis genes in Arabidopsis and Switchgrass.

**Dataset S2** Co‐expression analysis of secondary wall‐related transcription factors in Arabidopsis and Switchgrass.

**Dataset S3** Gene expression profiling (FPKM) from internodes of switchgrass PvMYB4OX, PvMYB58/63OX, PvMYB42/85OX and PvWRKY12DR lines.

**Dataset S4** Gene expression profiling (FPKM) from tillers of switchgrass PvMYB4OX, PvMYB58/63OX, PvMYB42/85OX and PvWRKY12DR lines.

**Dataset S5** List of differentially expressed genes from internodes and tillers of switchgrass PvMYB4OX, PvMYB58/63OX, PvMYB42/85OX and PvWRKY12DR lines compared with their equivalent control (*P* < 0.05).

**Dataset S6** List of genes that are both co‐expressed with PvWRKY12 and differentially expressed in the PvWRKY12DR line.

**Dataset S7** Comparison between the present QUBIC‐based method and the PCC method to detect TFs co‐expressed with switchgrass F5H in microarray data sets.
