# Supplementary material for: Gene regulatory networks for lignin biosynthesis in switchgrass (Panicum virgatum)
Source: Plant Biotechnol J. 2018 Sep 17;17(3):580–93. doi: 10.1111/pbi.13000 (PMC6381781; doi:10.1111/pbi.13000)
Supplement: Supplementary file 1 — Figure S1 Phylogenetic trees of MYB, NAC, bHLH, ERF and WRKY TFs that appear in Arabidopsis and switchgrass co‐expression networks of lignin biosynthesis. Figure S2 Co‐expression network of MYB and NAC TFs with lignin biosynthesis genes in Arabidopsis and switchgrass. Figure S3 Phylogenetic tree of MYB58/63 and MYB42/85 orthologs from Arabidopsis, Medicago, poplar, switchgrass, maize, rice, sorghum and Brachypodium. Figure S4 In situ hybridization of PvMYB58/63C and PvMYB42/85C in switchgrass stem. Figure S5 Additional in situ hybridization images for MYB58/63A. Figure S6 qRT‐PCR analysis of target transcripts in leaf of PvMYB58/63‐RNAi and PvMYB42/85‐RNAi transgenic switchgrass. Figure S7 qRT‐PCR analysis of lignin biosynthesis genes in leaf of PvMYB58/63A‐OX transgenic switchgrass. Figure S8 Phenotype of PvMYB58/63‐RNAi transgenic lines. Figure S9 Additional in situ hybridization images for MYB42/85A. Figure S10 Phenotypes of additional PvMYB42/85‐OX29, OX4 and OX1 plants in comparison to control lines. Figure S11 Lignin staining for PvMYB42/85 lines. Figure S12 qRT‐PCR analysis of lignin biosynthesis genes in leaf of PvMYB42/85A‐OX transgenic switchgrass. Figure S13 Phenotype of PvMYB42/85‐RNAi transgenic lines. Figure S14 Additional in situ hybridization images for WRKY 12. Figure S15 Stem outer diameters and stem radial thickness in PvWRKY12‐DR transgenic lines. Figure S16. Phenotype of PvSWN2‐RNAi transgenic lines. Figure S17 Phylogenetic tree of NST and SND2 orthologs from Arabidopsis, poplar, maize, rice and switchgrass. Figure S18 Cell wall‐related gene expression and cell wall component analysis in PvSND2‐RNAi transgenic switchgrass. Figure S19 Phenotype of PvSND2‐RNAi transgenic lines. Figure S20 Functional distribution of genes co‐expressed with TFs in Arabidopsis and switchgrass. Figure S21 Correlation matrix of switchgrass transcriptomes determined by the Pairwise Pearson correlation coefficients (PCC) method. Figure S22 qRT‐PCR analysis of SWN1 and SW [file PBI-17-580-s008.pdf]

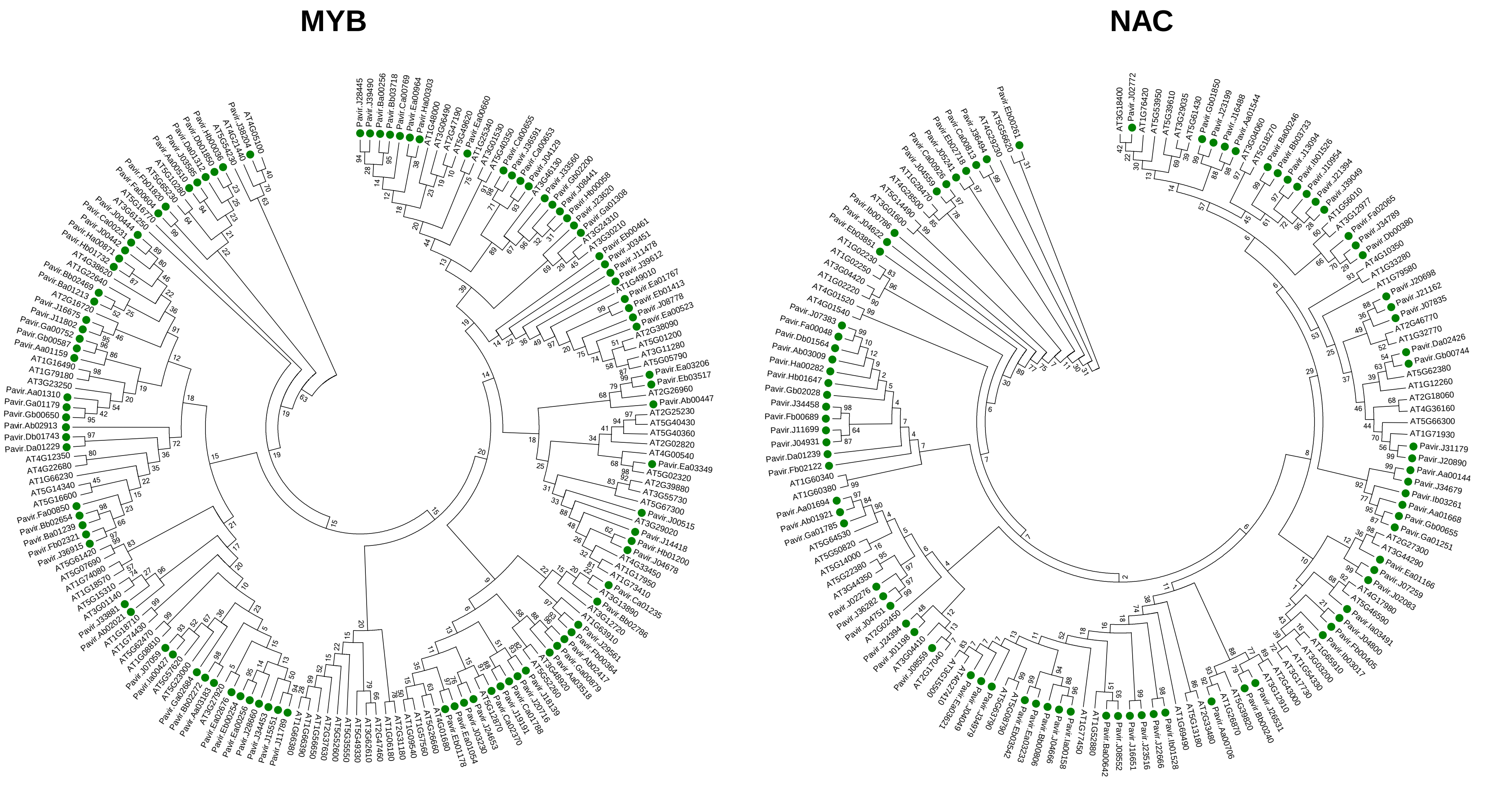

Supplementary Figure 1. Phylogenetic trees of MYB, NAC, bHLH, ERF and WRKY TFs that appear in Arabidopsis and switchgrass co-expression networks of lignin biosynthesis. Green dots indicate genes from Arabidopsis. The trees were created by the maximum parsimony algorithm using MEGA 5 with 95% partial deletion. The number at the node indicates percentage from 1,000 bootstraps.

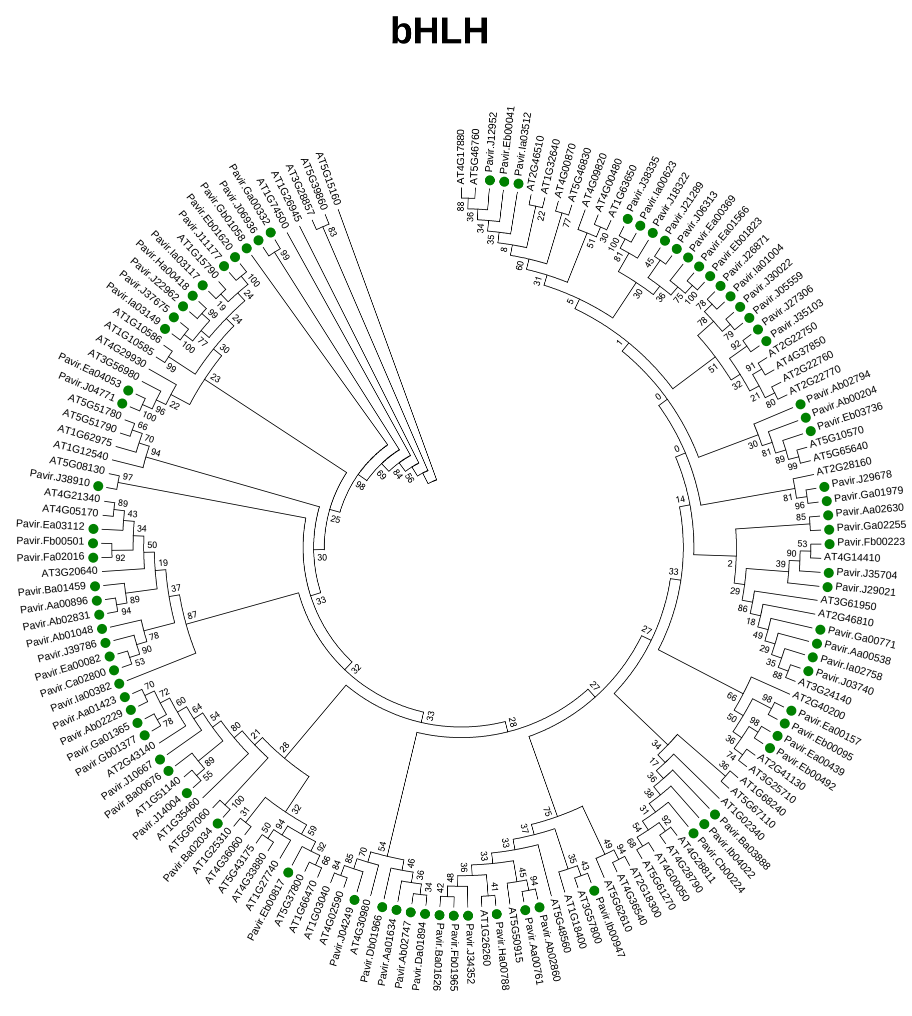

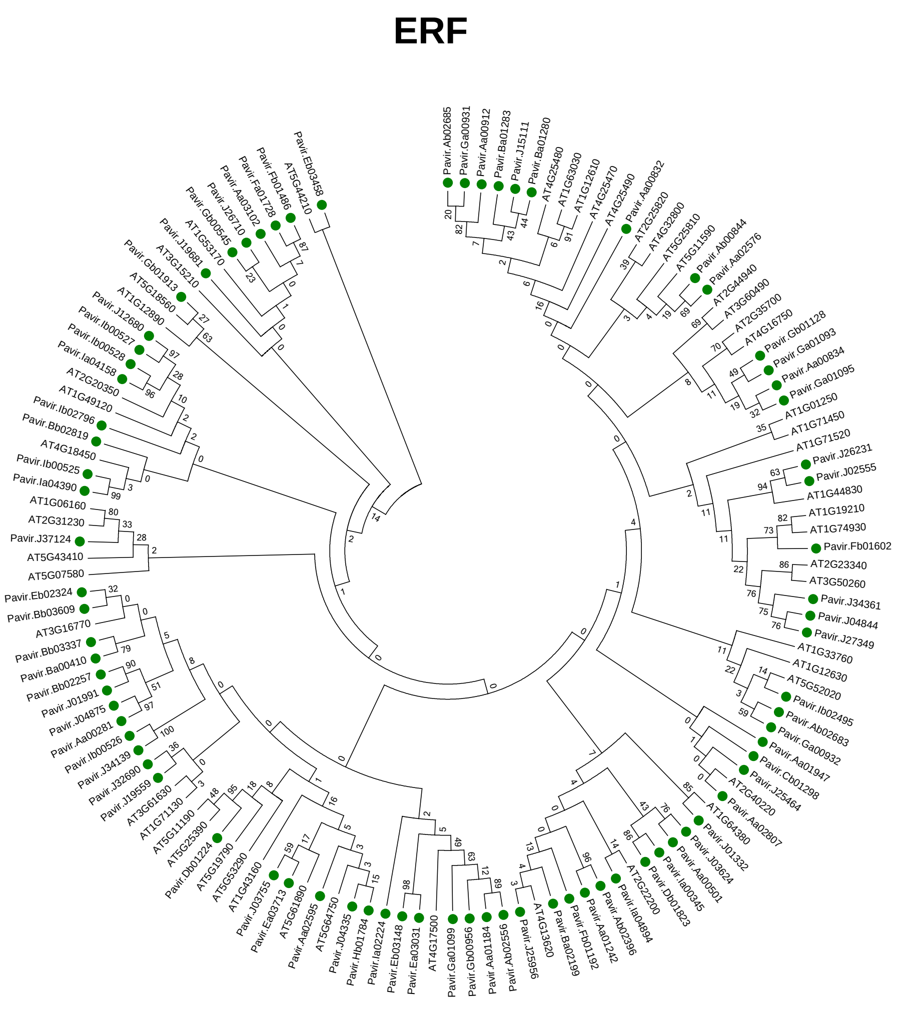

Supplementary Figure 1. (continued)

WRKY

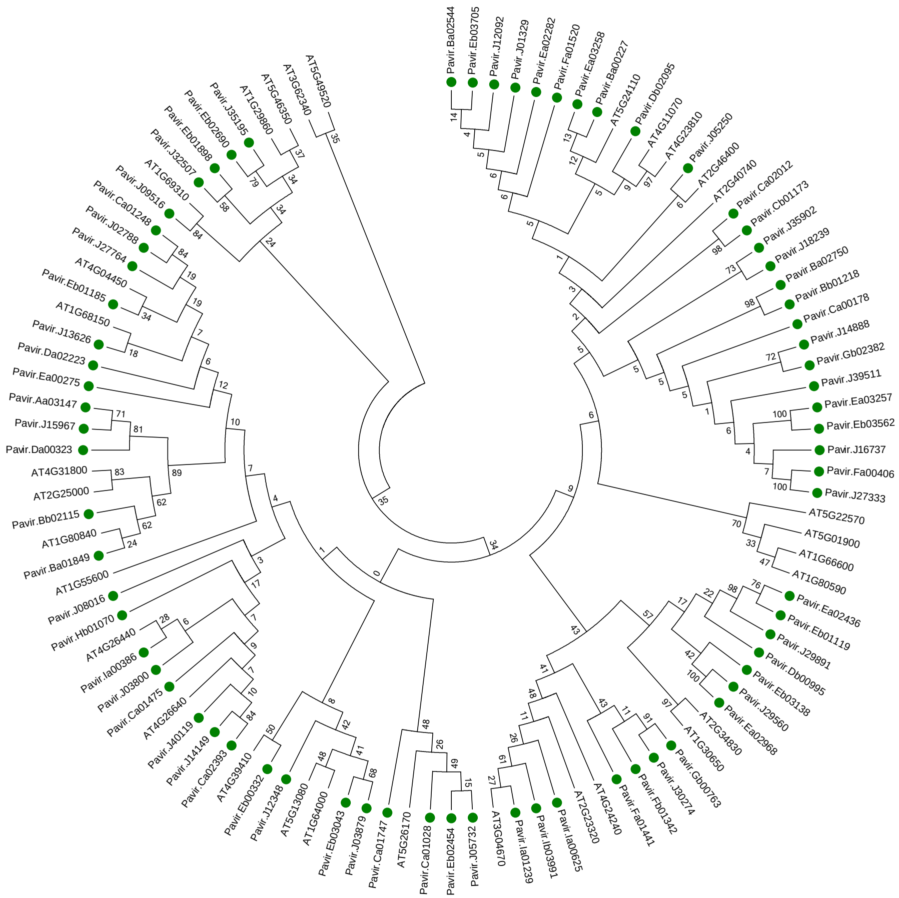

Supplementary Figure 1. (continued)

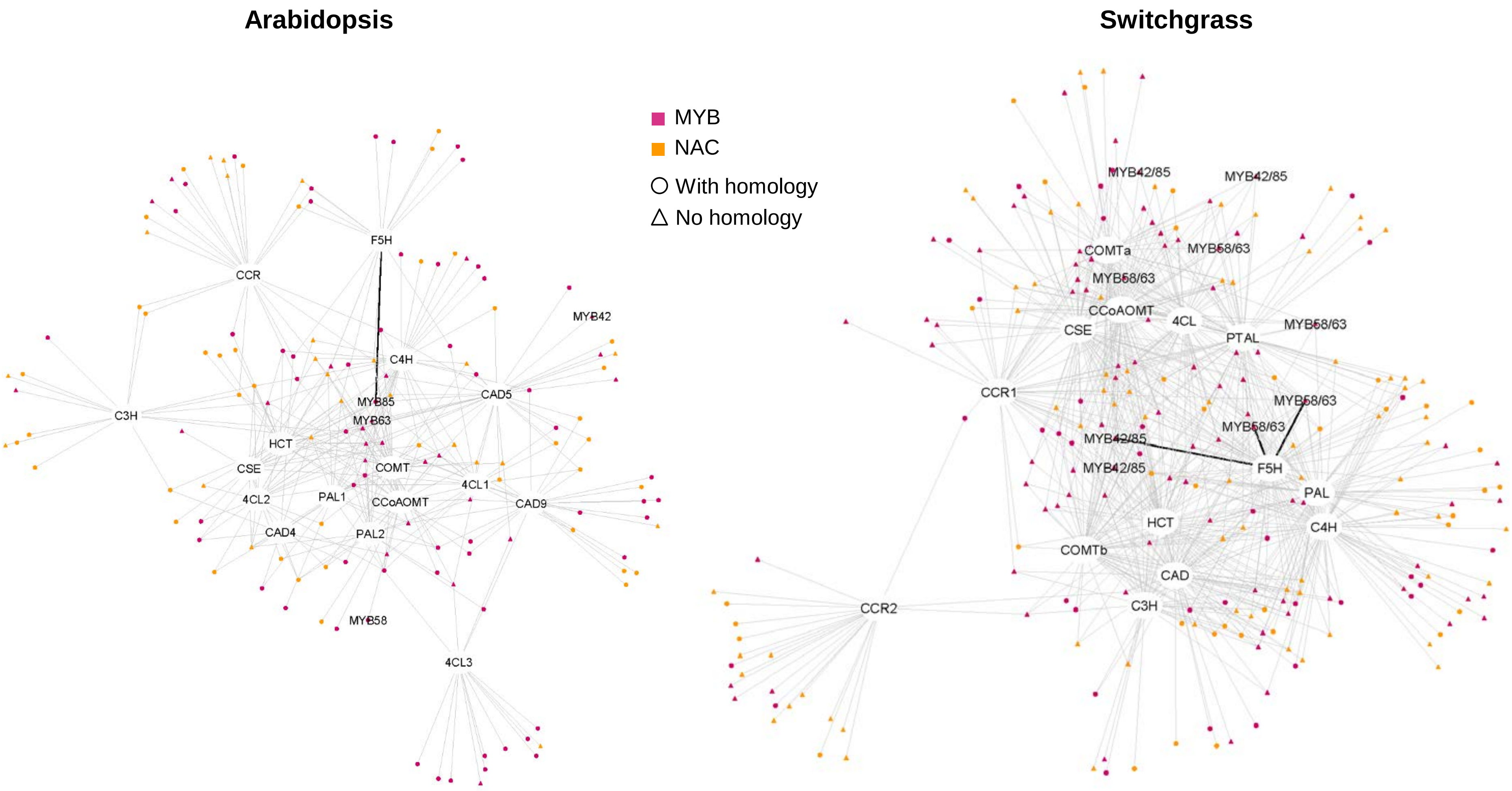

Supplementary Figure 2. Co-expression network of MYB and NAC TFs with lignin biosynthesis genes in Arabidopsis and switchgrass. Purple and orange colors indicate MYB- and NAC-type TFs, respectively. Circles indicate homologs in Arabidopsis and switchgrass. The correlation relationship between F5H and MYB42/85 and MYB58/63 is presented as a thicker line.

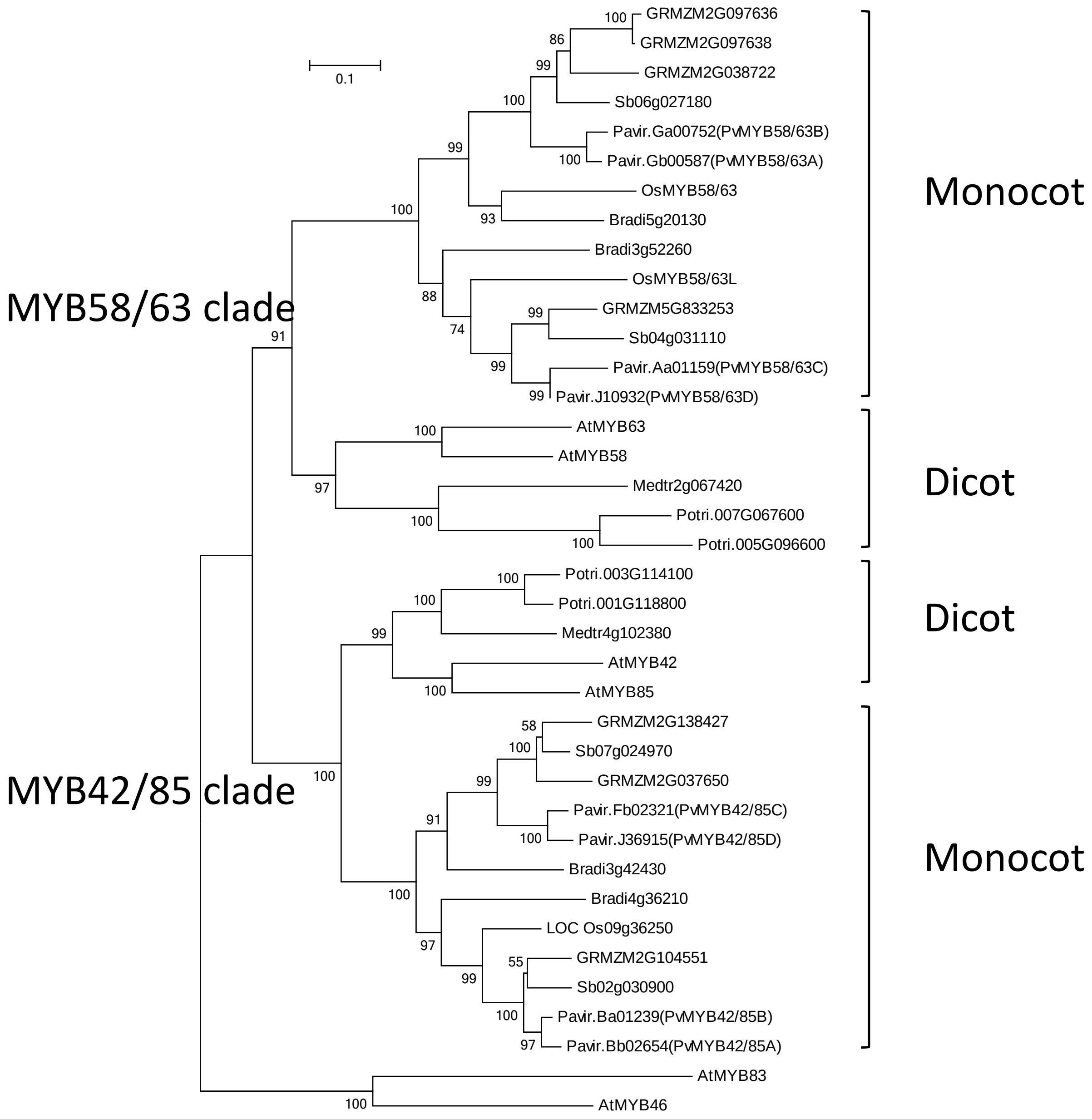

Supplementary Figure 3. Phylogenetic tree of MYB58/63 and MYB42/85 orthologs from Arabidopsis, Medicago, poplar, switchgrass, maize, rice, sorghum and *Brachypodium*. Arabidopsis MYB46 and MYB83 were set as outgroup. The trees were created by the neighbor-joining algorithm using MEGA 6. The number at the node indicates percentage from 1,000 bootstraps.

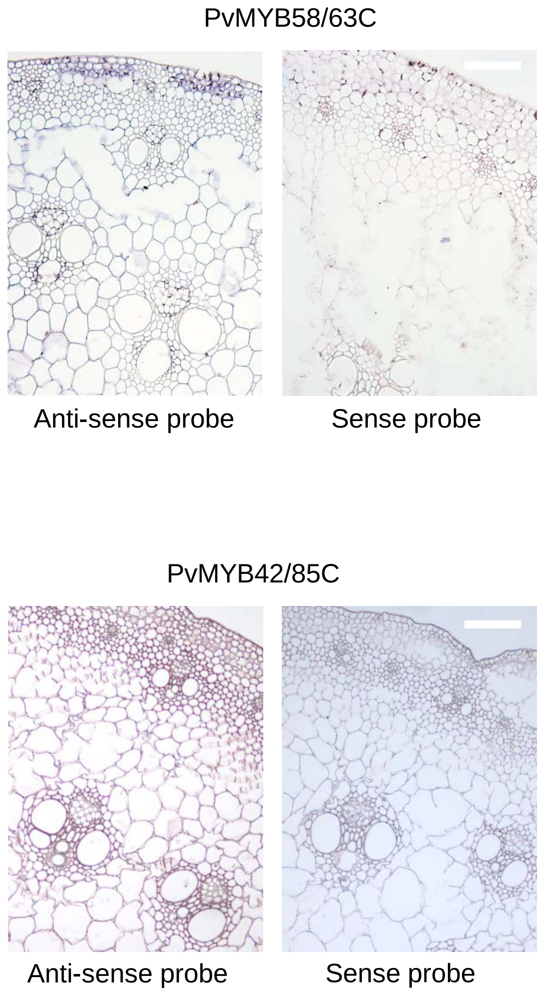

Supplementary Figure 4. In situ hybridization of PvMYB58/63C and PvMYB42/85C in switchgrass stems. Bars, 100 µm.

Supplementary Figure 5. Additional in situ hybridization images for MYB58/63A, E4 stage, internode 2.

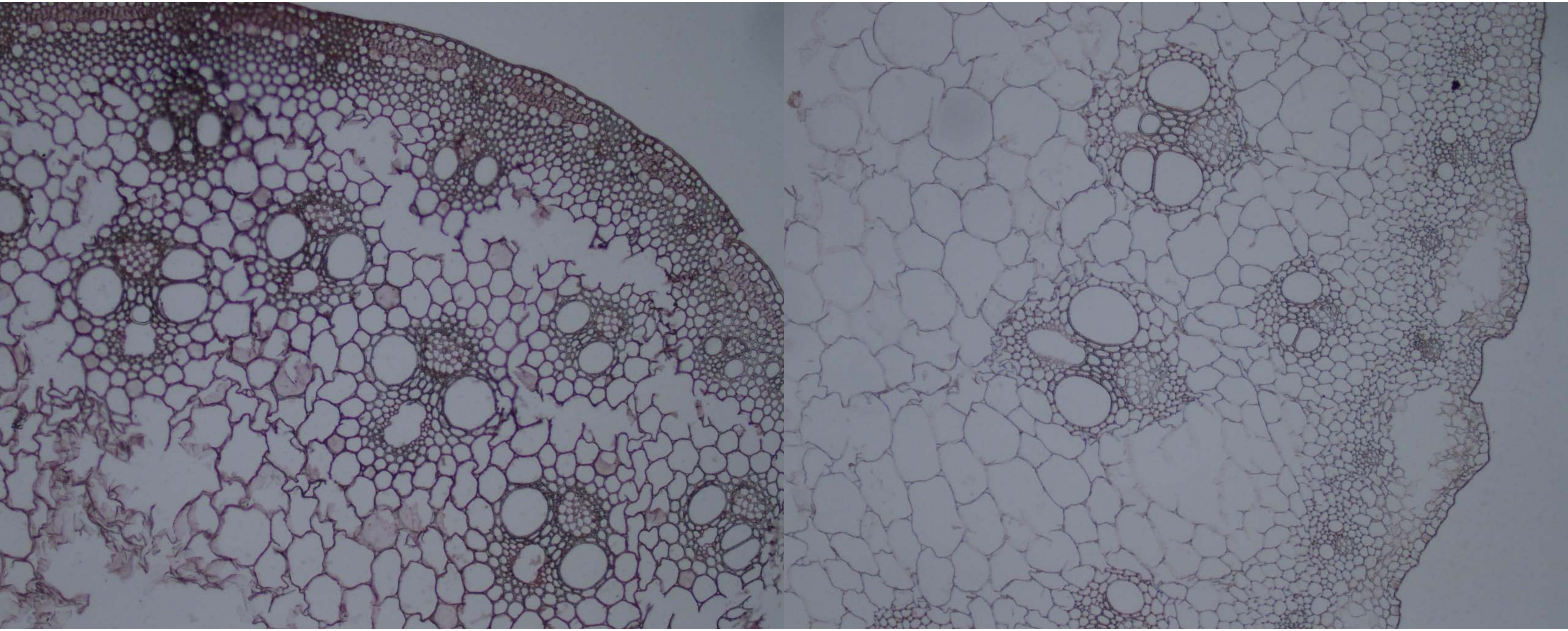

Anti-sense probe

Sense probe

A

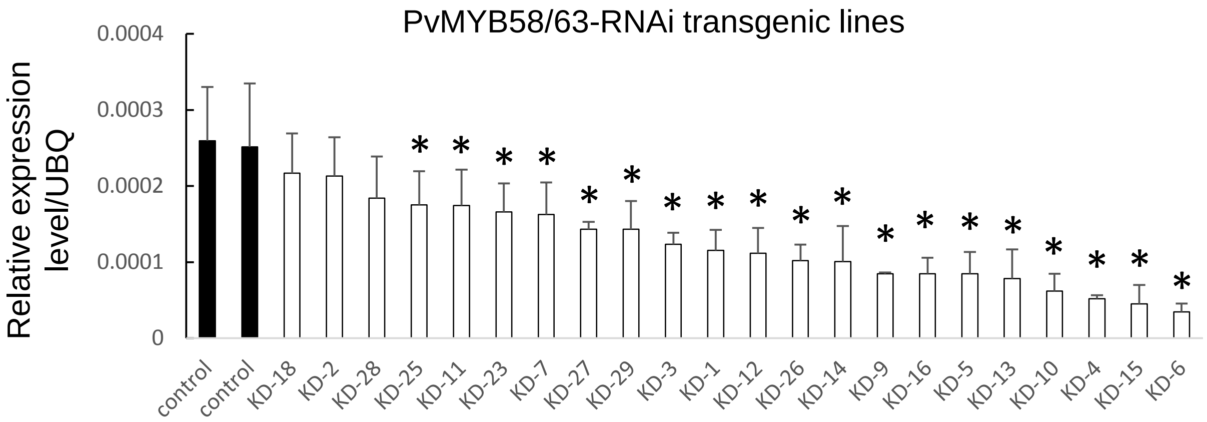

B

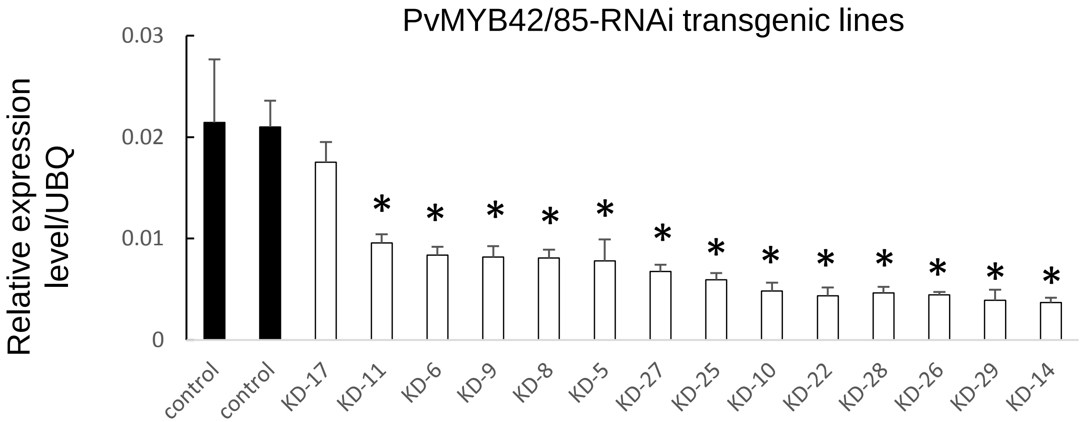

Supplementary Figure 6. qRT-PCR analysis of target transcripts in leaves of PvMYB58/63-RNAi (A) and PvMYB42/85-RNAi (B) transgenic switchgrass. KD, knockdown. Error bars represents the SE of three biological replicates. Asterisks on the tops of the bars indicate values significantly different from the control ($p < 0.05$) determined by the Student's *t*-test.

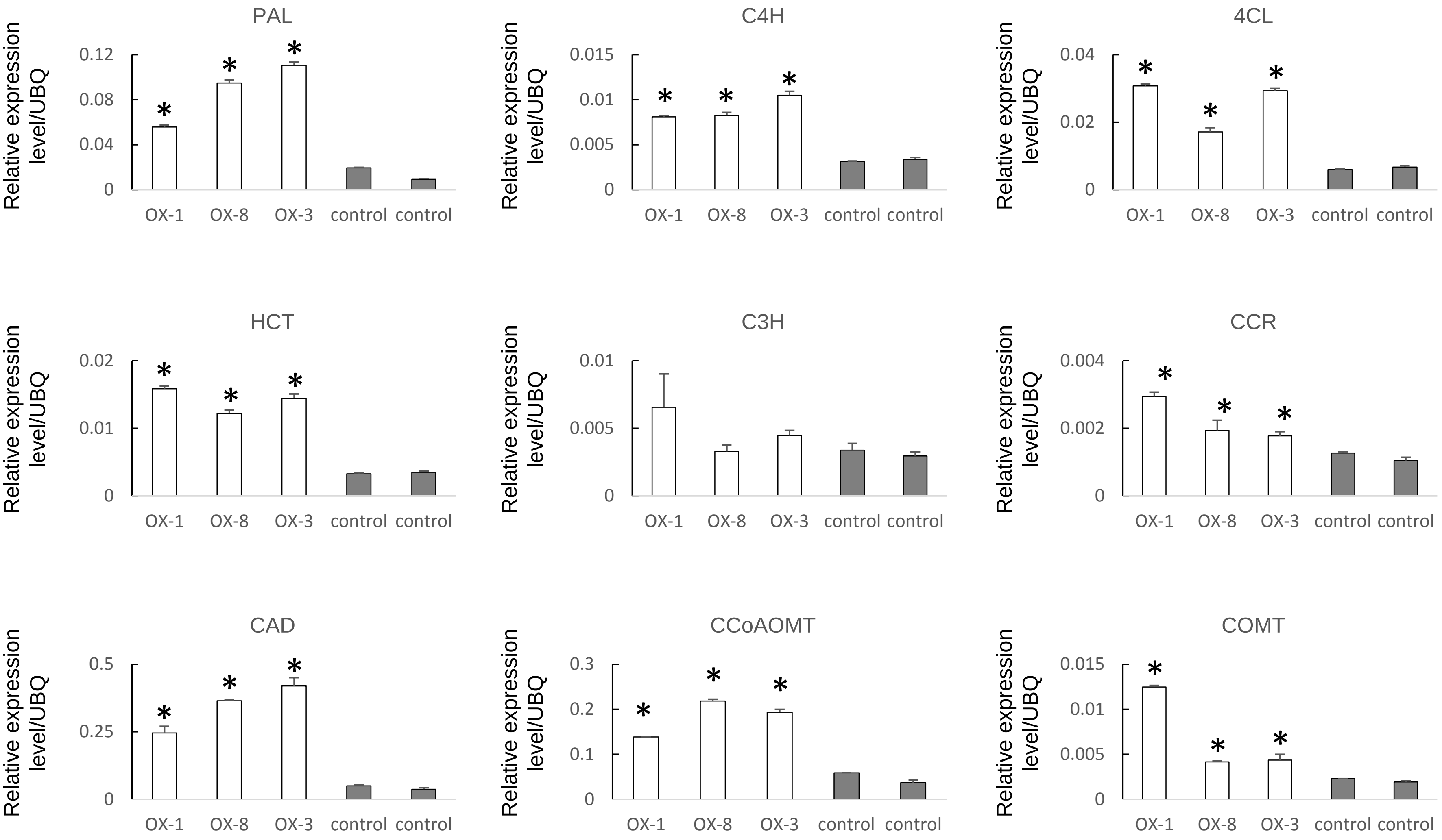

Supplementary Figure 7. qRT-PCR analysis of lignin biosynthesis genes in leaves of PvMYB58/63A-OX transgenic switchgrass. Error bars represents the SE of three biological replicates. Asterisks on the top of the bars indicate values significantly different from the control ($p < 0.05$) determined by the Student's *t*-test.

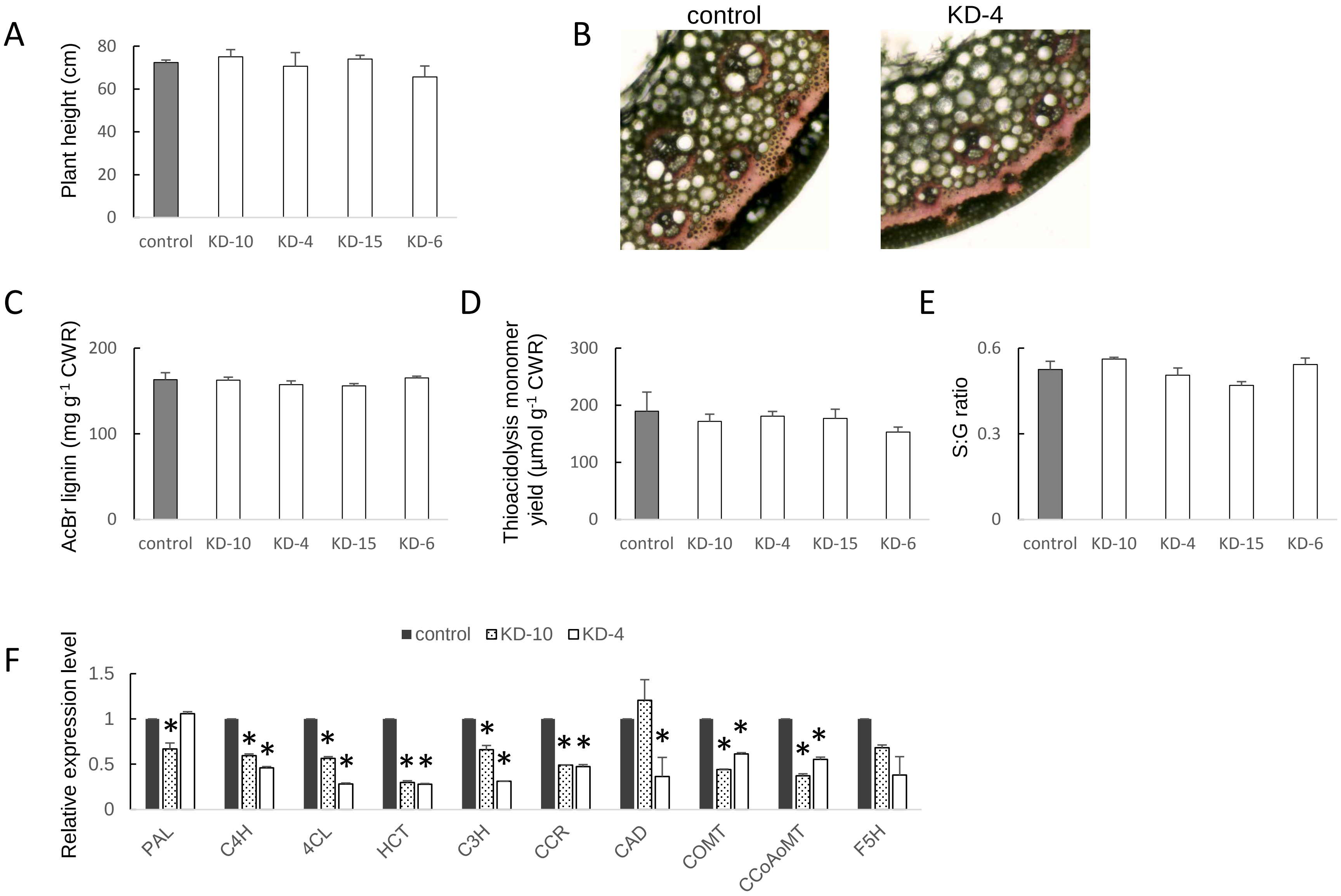

Supplementary Figure 8. Phenotypes of PvMYB58/63-RNAi transgenic lines. (A) Plant height. (B) Phloroglucinol-HCl staining of internode cross-sections. (C-D) Total lignin content determined by the AcBr (C) and thioacidolysis (D) methods. (E) Lignin S:G ratios. (F) qRT-PCR analysis of lignin biosynthesis gene transcripts in leaves. The transcript level of lignin biosynthesis genes in leaves from the control line was set to 1. Error bars represents the SE of three biological replicates. Asterisks on the tops of the bars indicate significant differences from the control ($p < 0.05$).

Supplementary Figure 9. Additional in situ hybridization images for MYB42/85A, E4 stage, internode 2.

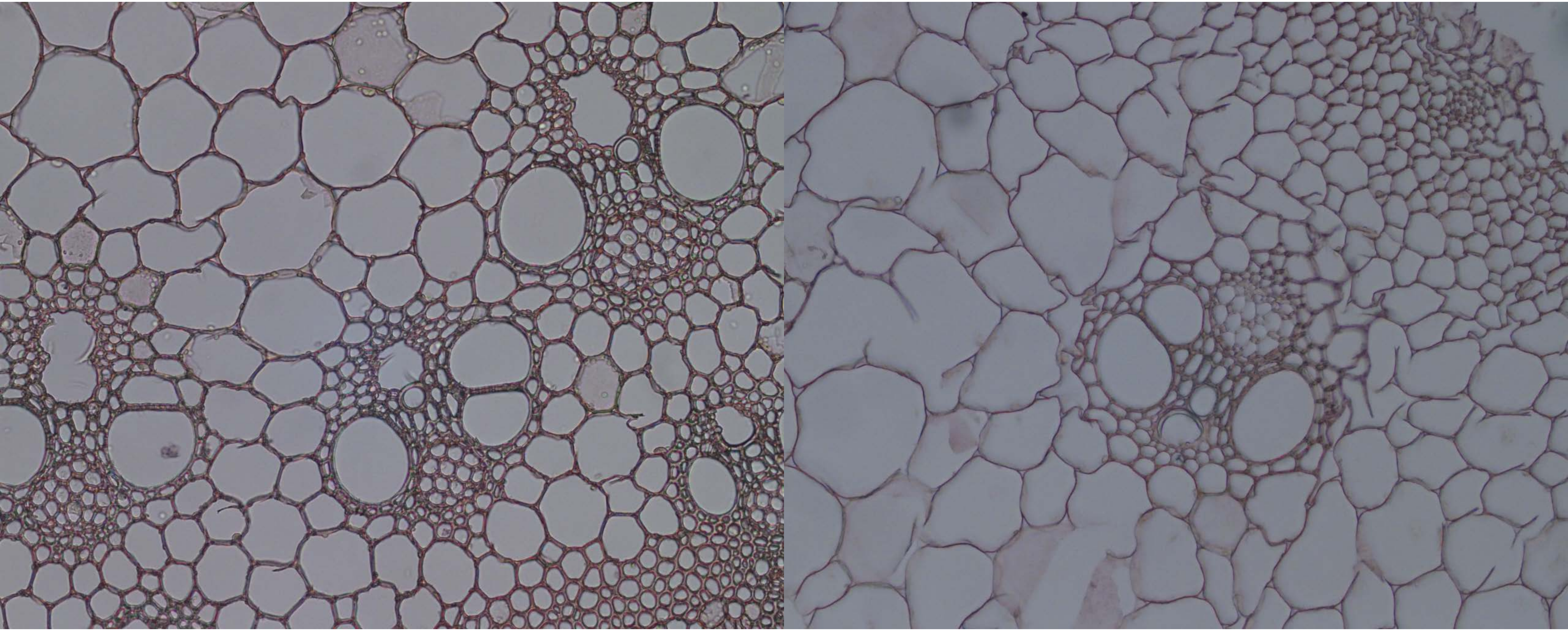

Anti-sense probe

Sense probe

Supplementary Figure 10. Phenotypes of additional PvMYB42/85-OX29, OX4 and OX1 plants in comparison to control lines.

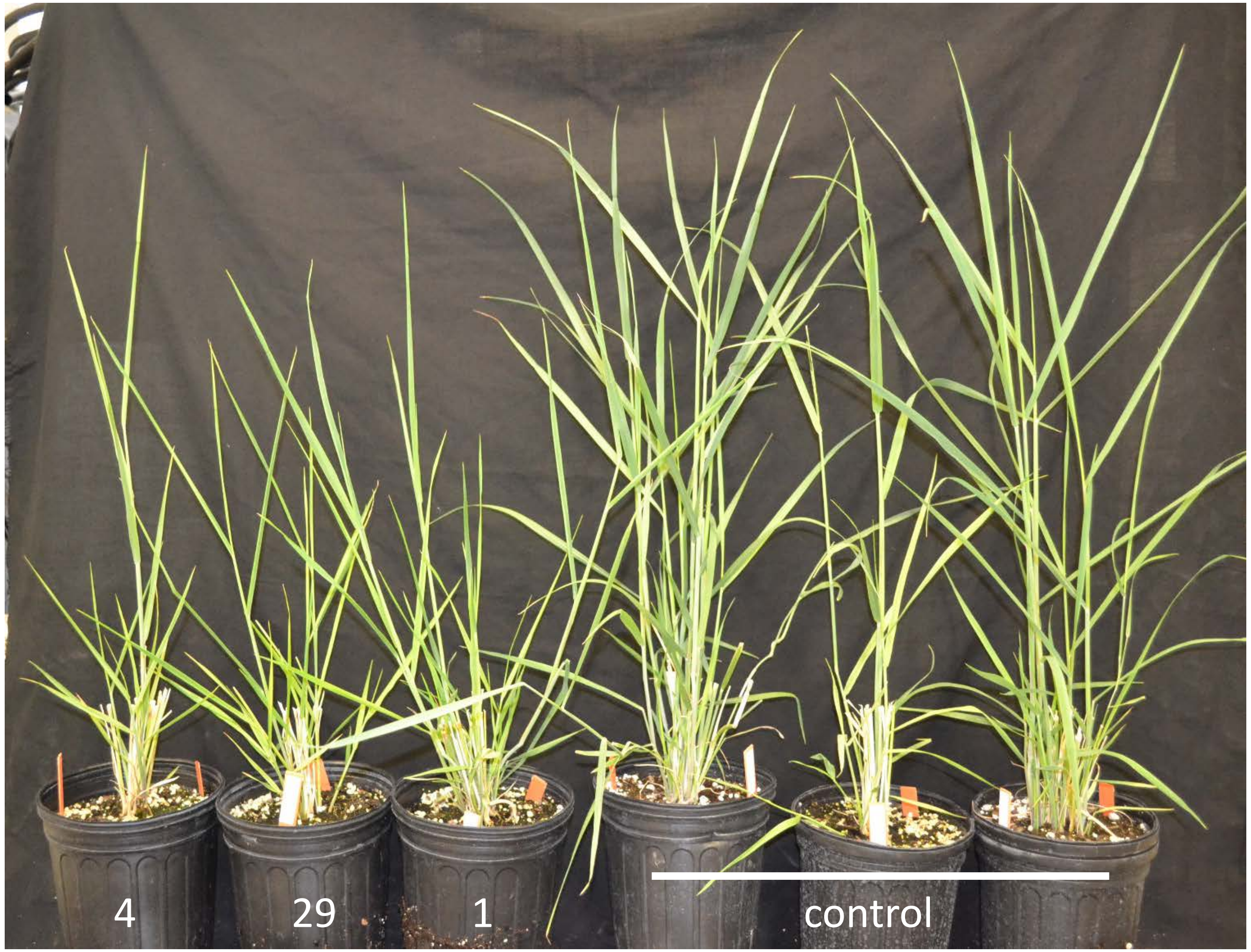

Supplementary Figure 11. Lignin staining for PvMYB42/85 lines (internode 2 at the E2 stage).

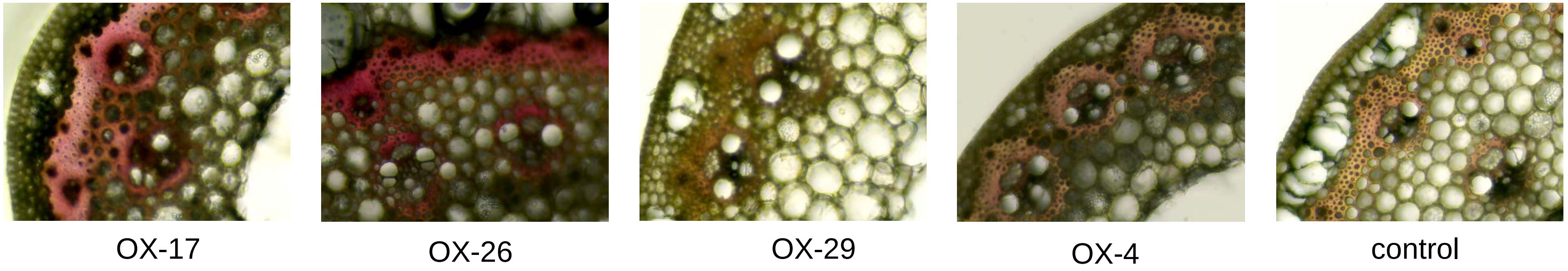

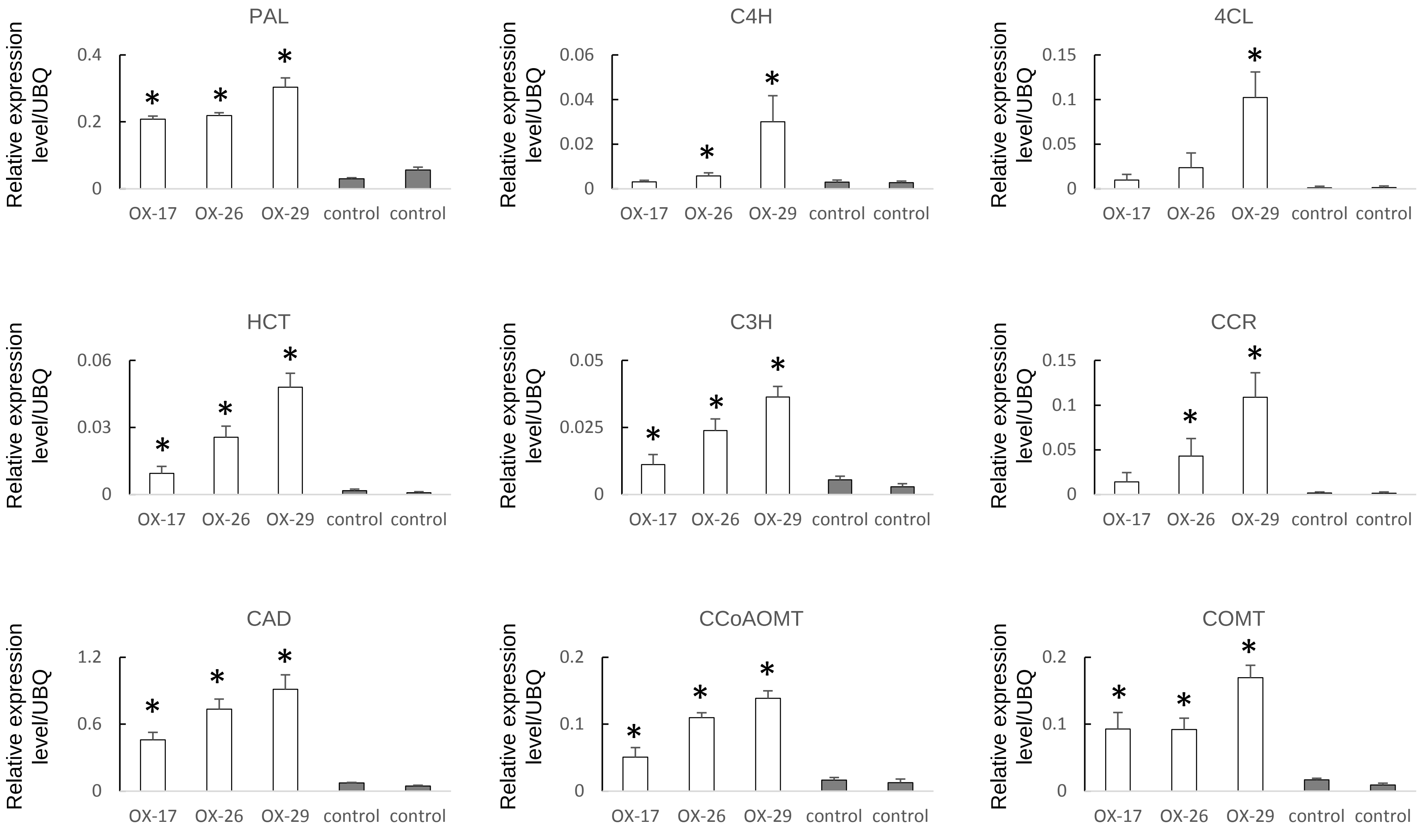

Supplementary Figure 12. qRT-PCR analysis of lignin biosynthesis genes in leaves of PvMYB42/85A-OX transgenic switchgrass. Error bars represents the SE of three biological replicates. Asterisks on the tops of the bars indicate significant differences from the control ($p < 0.05$).

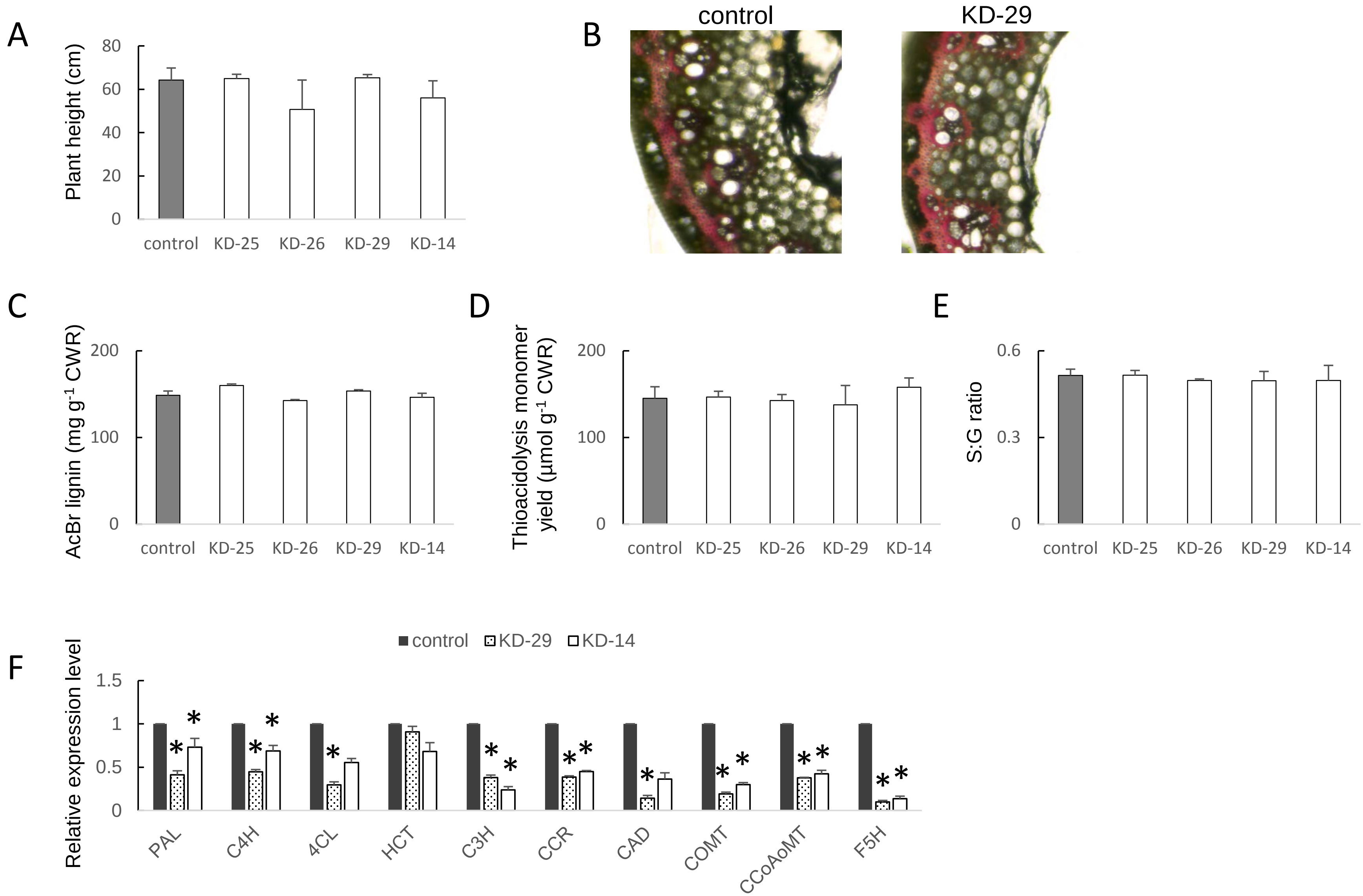

Supplementary Figure 13. Phenotypes of PvMYB42/85-RNAi transgenic lines. (A) Plant height. (B) Phloroglucinol-HCl staining of internode cross-sections. (C-D) Total lignin content determined by the AcBr (C) and thioacidolysis (D) methods. (E) Lignin S:G ratio.s (F) qRT-PCR analysis of lignin biosynthesis gene transcripts in leaves. The transcript levels of lignin biosynthesis genes in leaves from the control line was set to 1. Error bars represents the SE of three biological replicates. Asterisks on the tops of the bars indicate significant differences from the control (p < 0.05).

Supplementary Figure 14. Additional in situ hybridization images for WRKY 12, E4 stage, internode 2.

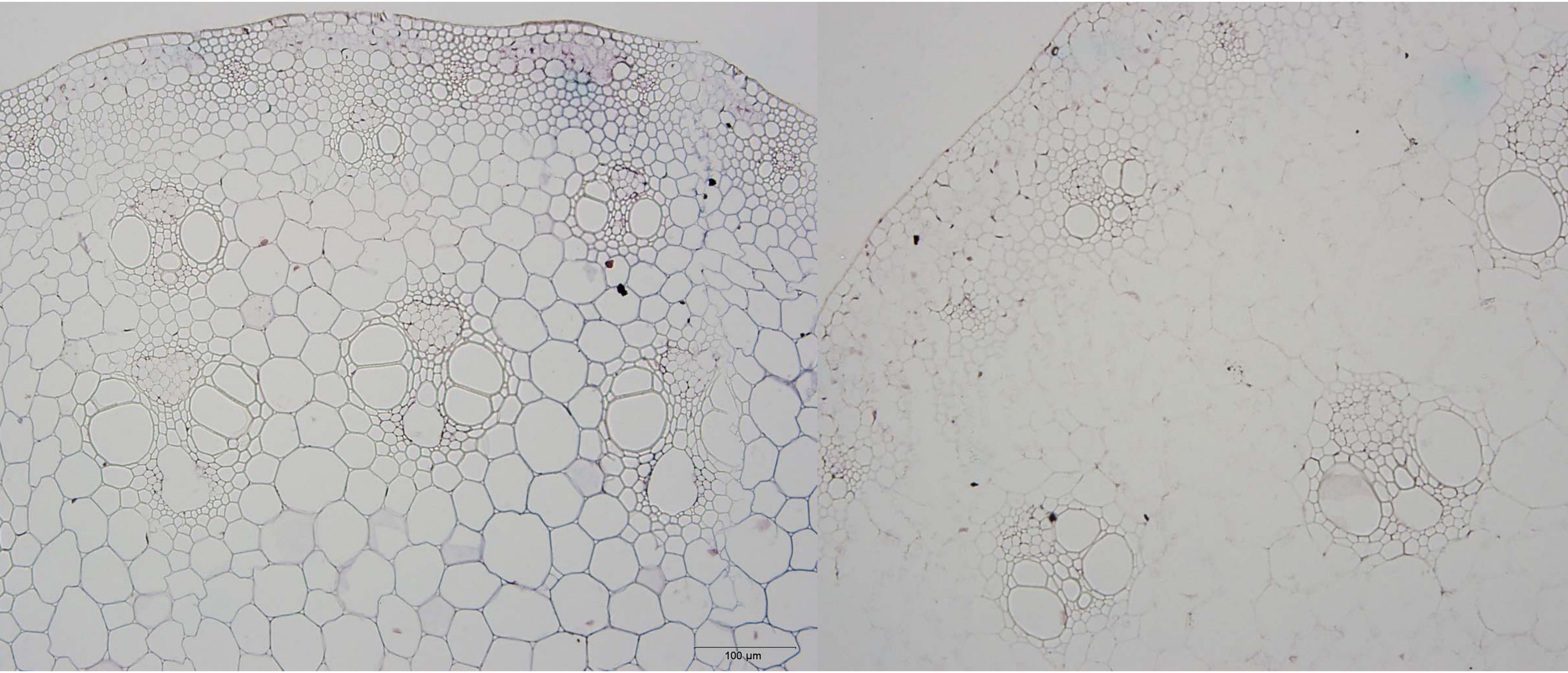

Anti-sense probe

Sense probe

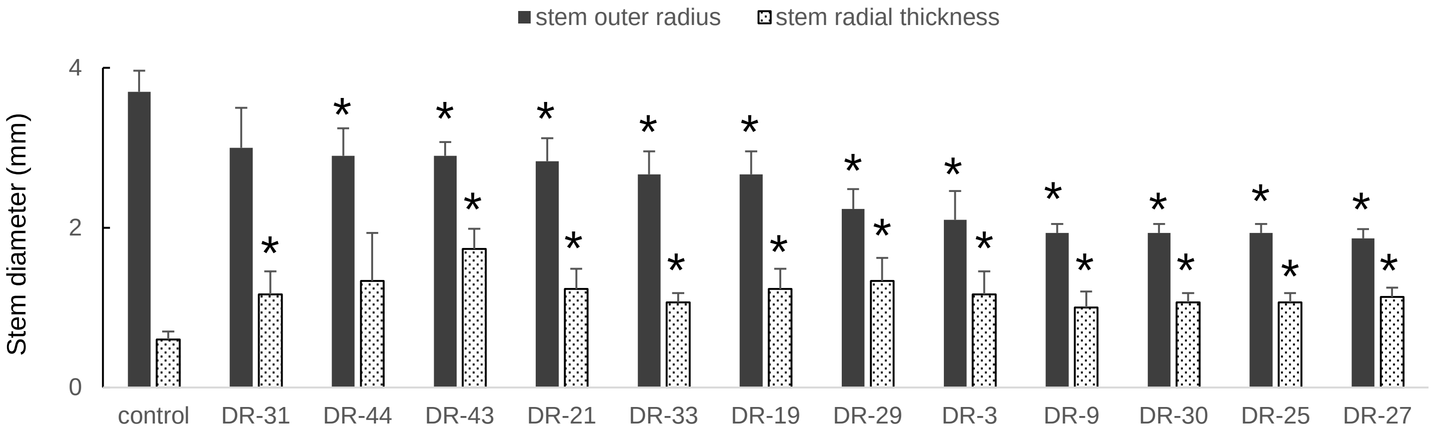

Supplementary Figure 15. Stem outer diameters and radial thickness in the PvWRKY12-DR transgenic line. Measurement was conducted on internode 2 of E4 tillers. Error bars represents the SE of at least three biological replicates. Asterisks on the tops of the bars indicate significant differences from the control ($p < 0.05$).

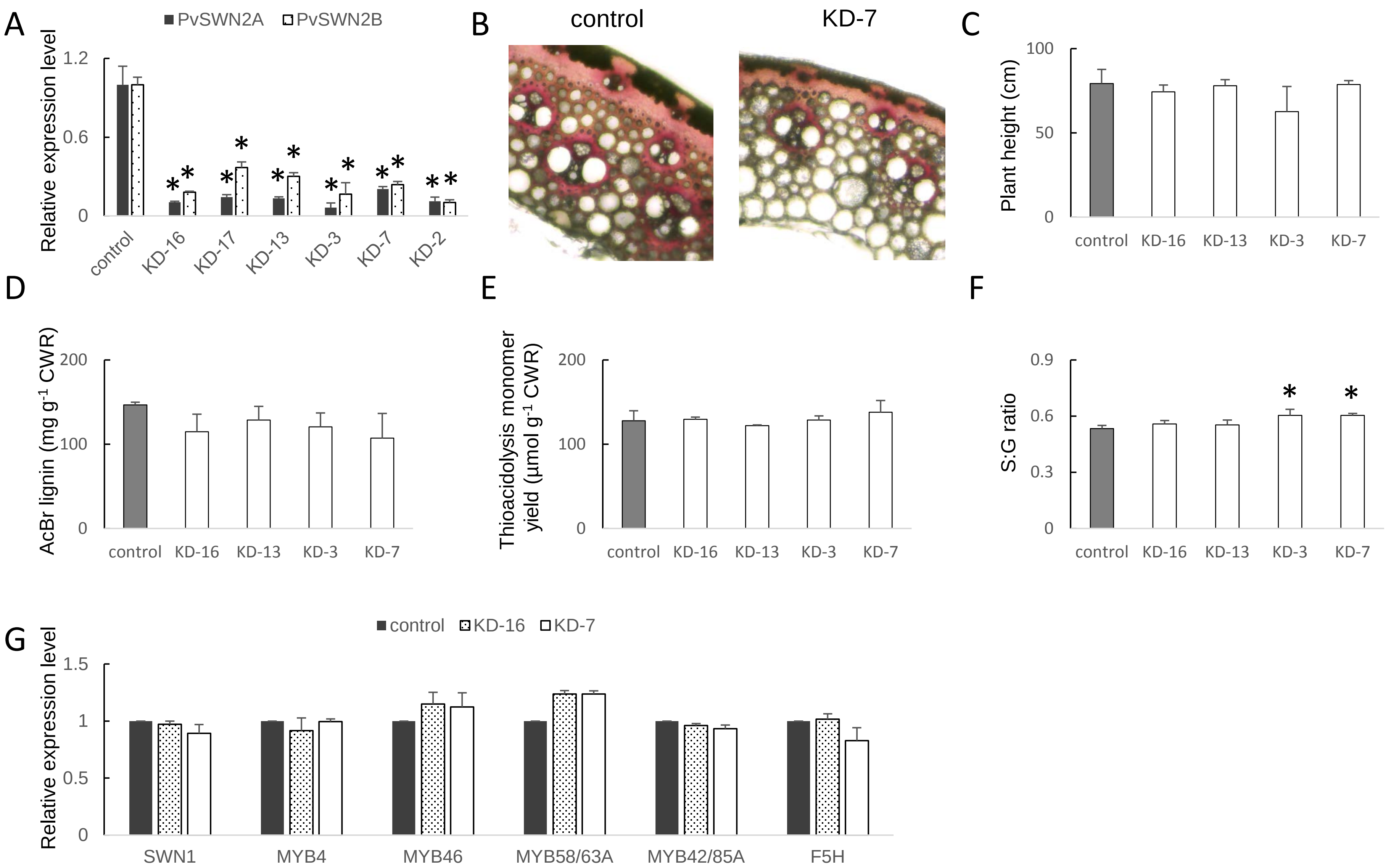

Supplementary Figure 16. Phenotypes of PvSWN2-RNAi transgenic lines. (A) qRT-PCR analysis of PvSWN2A and PvSWN2B in leaves. (B) Phloroglucinol-HCl staining of internode cross-sections. (C) Plant height. (D-E) Total lignin content determined by the AcBr (D) and thioacidolysis (E) methods. (F) Lignin S:G ratios. (G) qRT-PCR analysis of lignin biosynthesis gene transcripts in leaves. For qRT-PCR (A) and (G), the transcript levels of the target genes in leaves from the control line was set to 1. Error bars represents the SE of three biological replicates. Asterisks on the tops of the bars indicate significant differences from the control ($p < 0.05$).

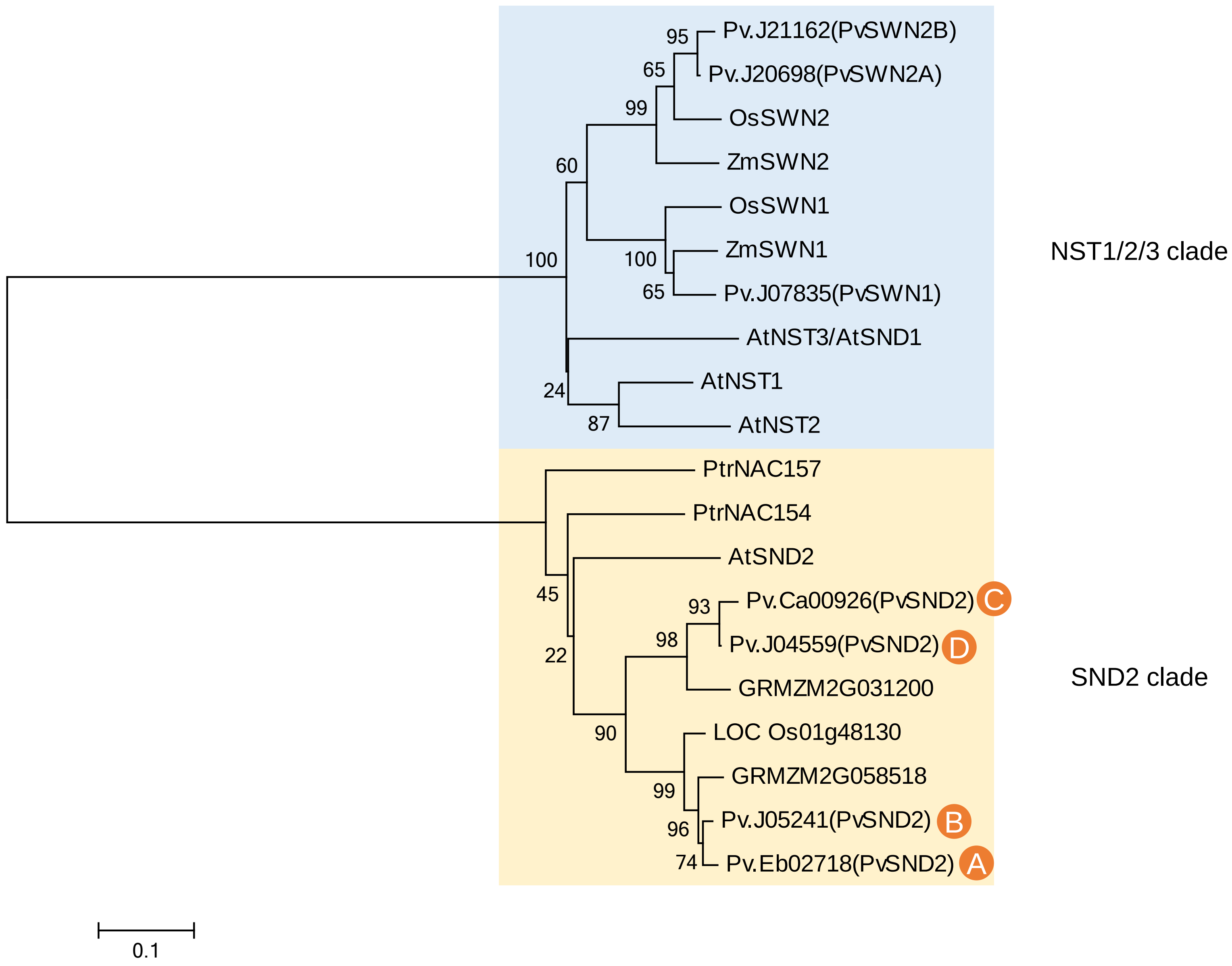

Supplementary Figure 17. Phylogenetic tree of NST and SND2 orthologs from Arabidopsis, poplar, maize, rice and switchgrass. The trees were created by the neighbor-joining algorithm using MEGA 6. The number at the node indicates percentage from 1,000 bootstraps.

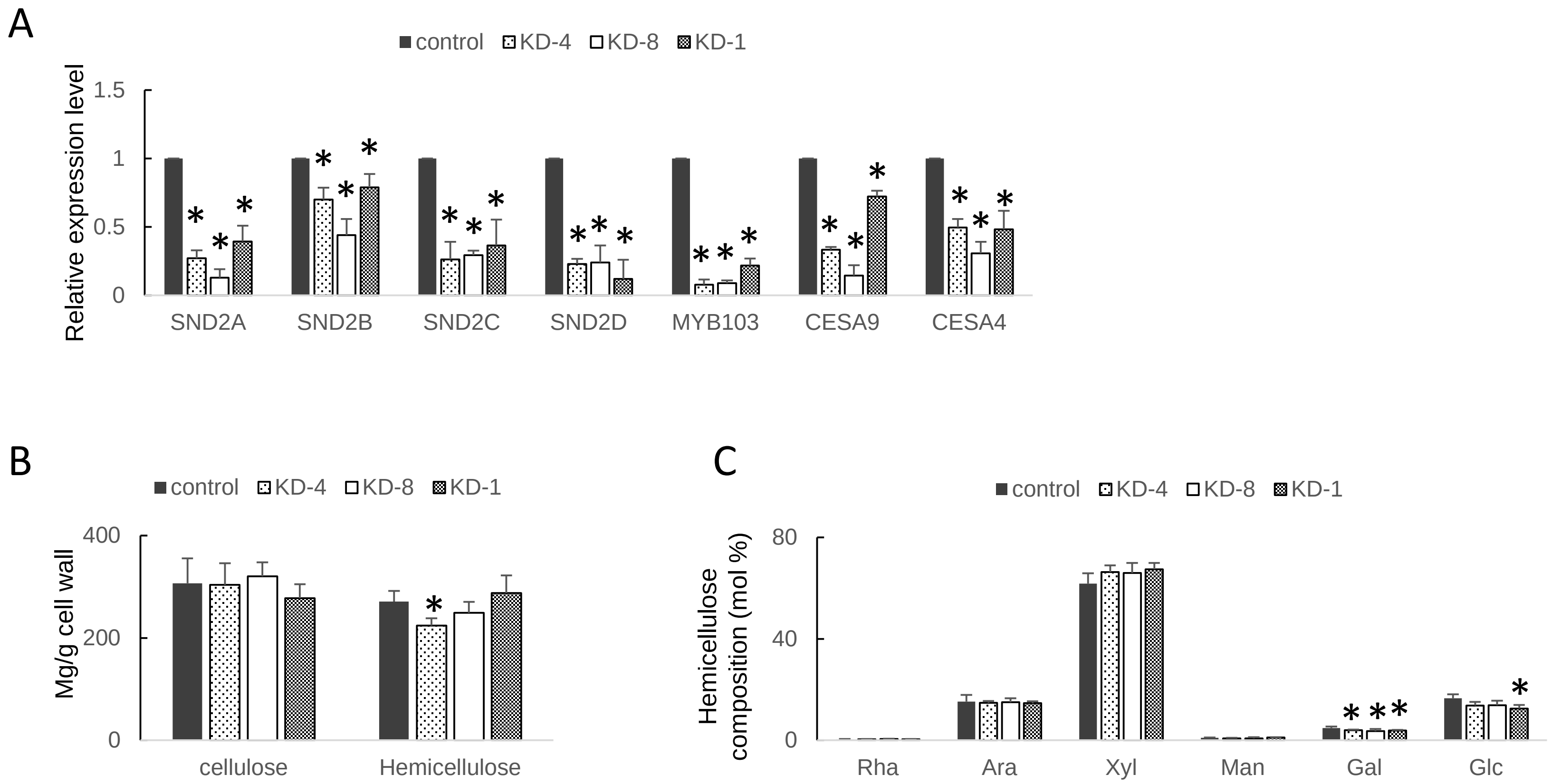

Supplementary Figure 18. Cell wall-related gene expression and cell wall fraction analysis in PvSND2-RNAi transgenic lines. (A) qRT-PCR analysis of SND2, MYB103, CESA7 and CESA8 transcripts in leaves. The expression level of target gene transcripts in leaves from the control line was set to 1. (B) Cellulose and hemicellulose content in tillers. (C) Neutral glycosyl residue composition of the hemicellulose fraction. Rha, rhamnose, Ara, Arabinose, Xyl, xylose, Man, mannose, Gal, galactose, and Glc, glucose. All data are means ± SE (n=3). Asterisks on the tops of the bars indicate significant differences from the control ($p < 0.05$).

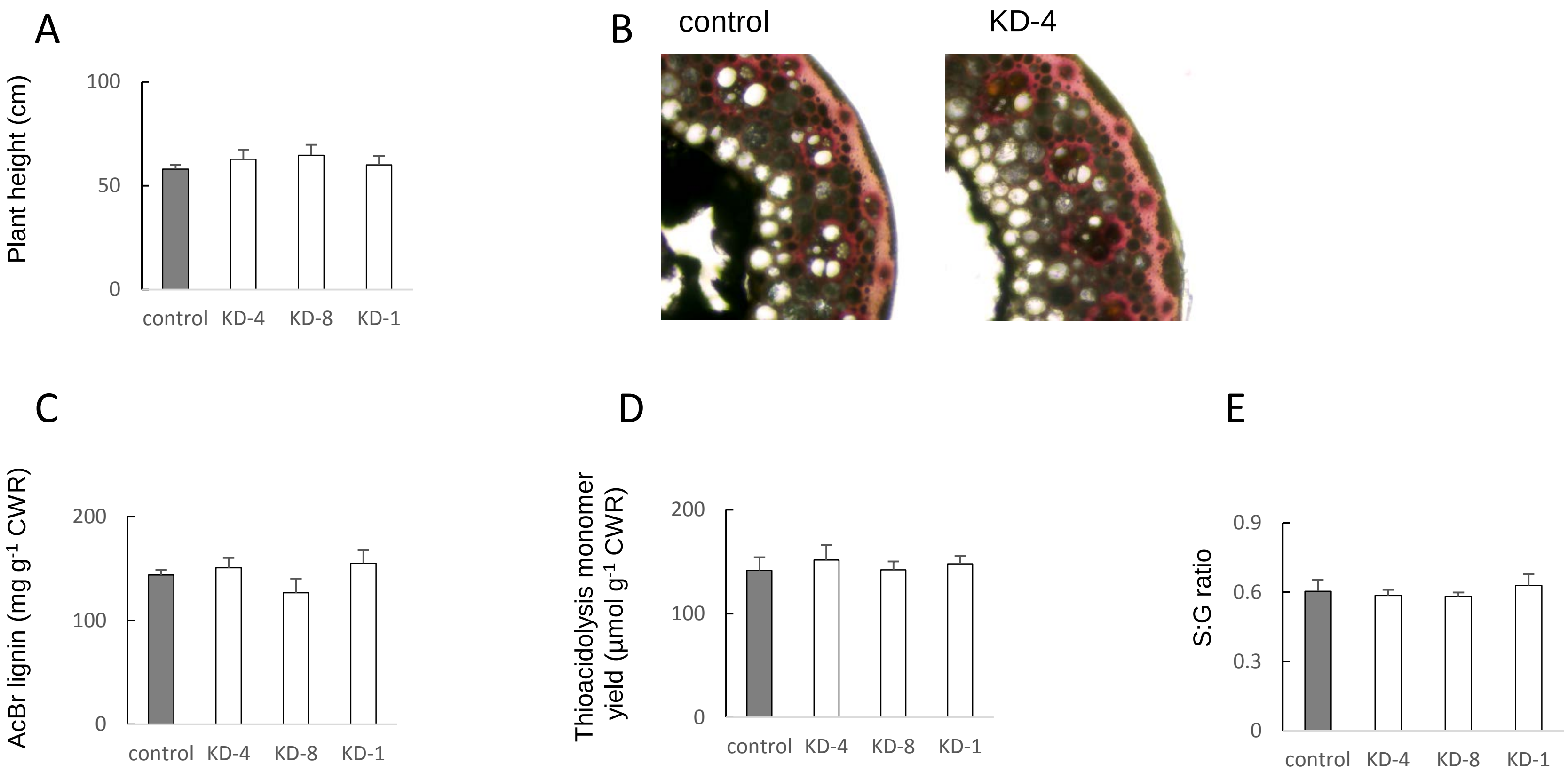

Supplementary Figure 19. Phenotypes of PvSND2-RNAi transgenic lines. (A) Plant height. (B) Phloroglucinol-HCl staining of internode cross-sections. (C-D) Total lignin content determined by the AcBr (C) and thioacidolysis (D) methods. (E) Lignin S:G ratios. Error bars represent the SE of three biological replicates.

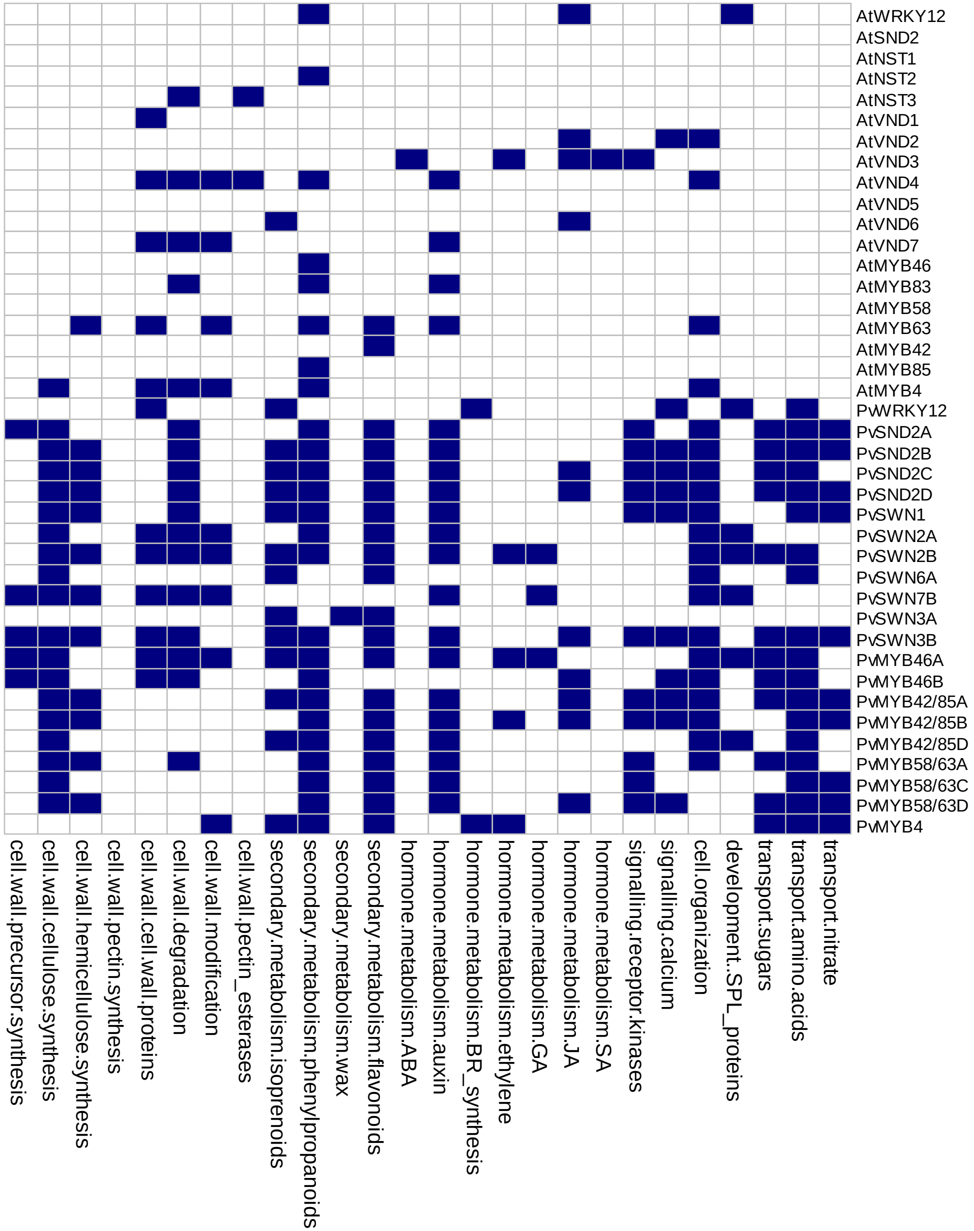

Supplementary Figure 20. Functional distribution of genes co-expressed with TFs in Arabidopsis and switchgrass. Blue color represents enriched functional groups identified by Fisher exact test (FDR<0.1).

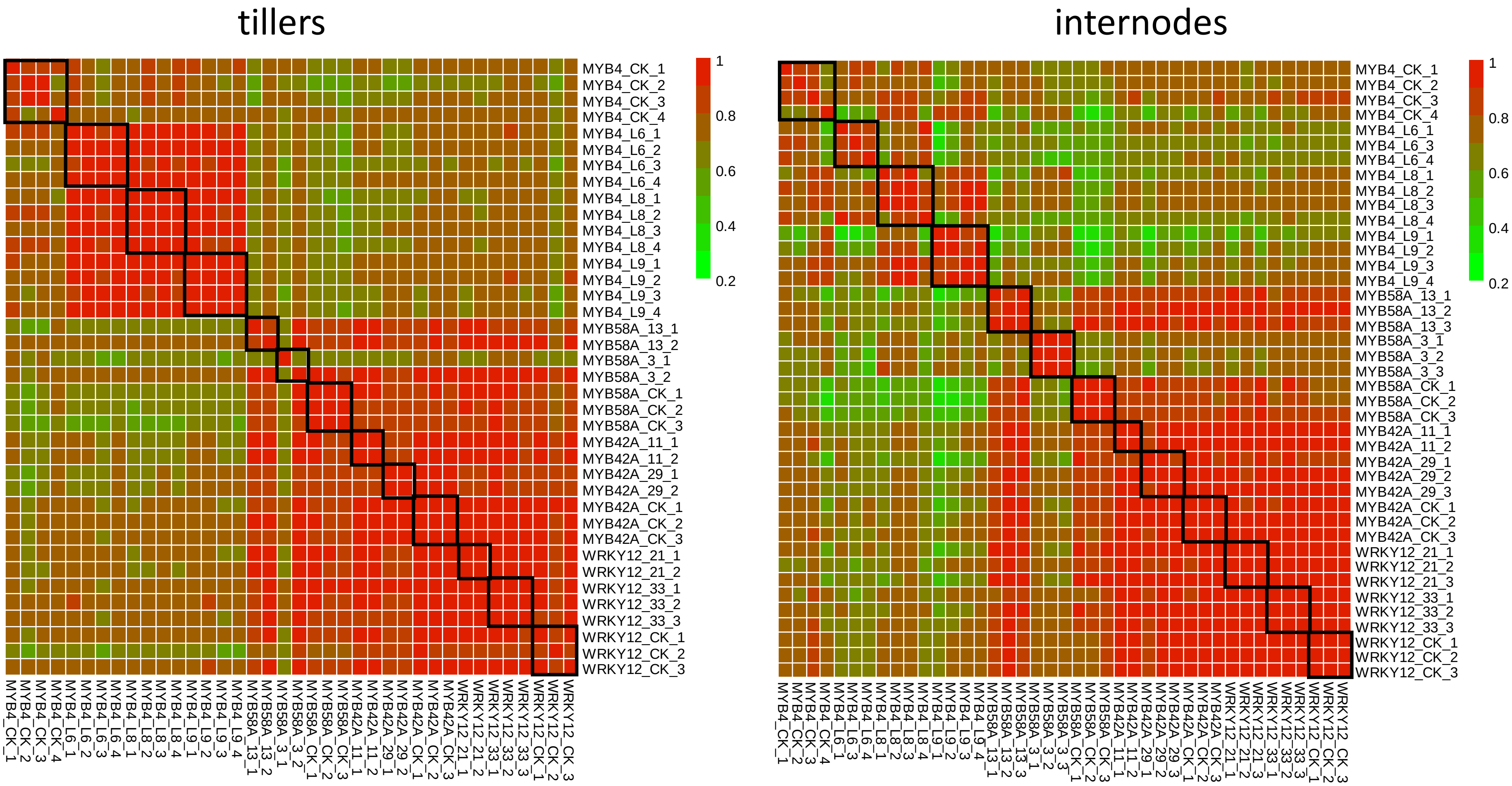

Supplementary Figure 21. Correlation matrix of switchgrass transcriptomes determined by the Pairwise Pearson correlation coefficients (PCC) method. The color scale indicates the degree of correlation.

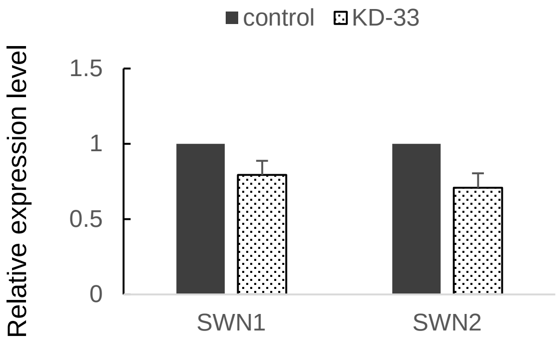

Supplementary Figure 22. qRT-PCR analysis of SWN1 and SWN2 transcripts in the WRKY12DR transgenic line compared to control. The transcript level of the target gene in tillers from the control line was set to 1.

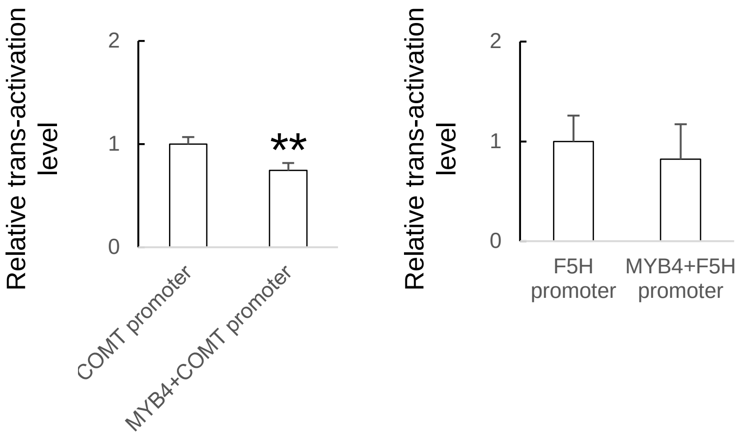

Supplementary Figure 23. Trans-activation of the PvCOMT and PvF5H promoters by PvMYB4. The activities in the protoplasts transfected with promoter-reporter construct and an empty effector construct are set to 1. All data are means ± SE (n = 3). Significant differences for the activation of PvCOMT and PvF5H promoters were determined by the Student's t test and are represented by a single asterisk ($p < 0.05$) or double asterisk ($p < 0.01$).

Supplementary Table 1. Lignin biosynthesis genes as bait genes for co-expression analysis in Arabidopsis and Switchgrass.

	**Arabidopsis**		**Switchgrass**	
Classifications	Name	Gene ID	Name	Probe ID
**PAL**	AtPAL1	At2g37040	PvPTAL	KanlowCTG00004_s_at
	AtPAL2	At3g53260	PvPAL	KanlowCTG00211_s_at
**C4H**	AtC4H	At2g30490	PvC4H	KanlowCTG11894_s_at
**4CL**	At4CL1	At1g51680	Pv4CL	KanlowCTG00833RC_s_at
	At4CL2	At3g21240		
	At4CL3	At1g65060		
**C3'H**	AtC3’H1	At2g40890	PvC3'H	AP13ITG41630_at
**CCoAOMT**	AtCCoAOMT1	At4g34050	PvCCoAOMT	KanlowCTG00900_s_at
**HCT**	AtHCT	At5g48930	PvHCT	AP13CTG44530_s_at
**F5H1**	AtF5H1	At4g36220	PvF5H	AP13ITG56842_at
**CCR**	AtCCR1	At1g15950	PvCCR1	KanlowCTG19403_s_at
			PvCCR2	AP13ITG69021_s_at
**COMT**	AtCOMT1	At5g54160	PvCOMTa	KanlowCTG00989_s_at
			PvCOMTb	AP13ITG62564_at
**CAD**	AtCAD4	At3g19450	PvCAD	VS16ITG06742_s_at
	AtCAD5	At4g34230		
	AtCAD9	At4g39330		
**CSE**	AtCSE	At1g52760	PvCSE	AP13ITG63270_at

Supplementary Table 2. Numbers of TFs of different families that are co-expressed with lignin biosynthesis genes in Arabidopsis and Switchgrass.

	TFs Co-expressed with lignin biosynthesis genes	
TF family	Arabidopsis	Switchgrass
AP2	10	17
ARF	3	23
ARR-B	3	6
B3	18	18
BBR-BPC	1	1
BES1	2	1
C2H2	37	66
C3H	9	36
CAMTA	0	15
CO-like	7	12
CPP	1	0
DBB	5	7
Dof	13	22
E2F/DP	0	3
EIL	3	7
ERF	51	102
FAR1	2	18
G2-like	12	34
GATA	9	18
GRAS	7	29
GRF	3	3
GeBP	8	6
HB-PHD	0	2
HB-other	0	7
HD-ZIP	19	29
HRT-like	0	0
HSF	6	23
LBD	18	16
LFY	1	0
LSD	1	8
M-type	16	2
MIKC	16	39
MYB	81	124
MYB_related	22	42
NAC	64	109
NF-X1	0	1
NF-YA	8	12

NF-YB	3	6
NF-YC	3	9
NZZ/SPL	0	0
Nin-like	7	7
RAV	1	5
S1Fa-like	0	2
SAP	1	0
SBP	7	18
SRS	4	1
STAT	0	1
TALE	13	44
TCP	5	9
Trihelix	3	19
VOZ	0	2
WOX	9	5
WRKY	31	90
Whirly	0	0
YABBY	0	1
ZF-HD	5	3
bHLH	70	111
bZIP	27	83
**Total**	**645**	**1274**

Supplementary Table 3. Secondary wall-related TFs as bait genes for co-expression analysis in Arabidopsis and Switchgrass.

	**Arabidopsis**		**Switchgrass**	
Classifications	Nick name	gene ID	Nick name	Probe ID
**WRKY12**	AtWRKY12	At2g44745	PvWRKY12	AP13ITG59374_at
**SND2**	AtSND2	At4g28500	PvSND2A	AP13ITG71892_at
			PvSND2B	AP13CTG15049_s_at
			PvSND2C	KanlowCTG43583_s_at
			PvSND2D	AP13ITG74807_s_at
**NST**	AtNST1	At2g46770	PvSWN1	AP13ITG69208_s_at
	AtNST2	At3g61910	PvSWN2A	AP13ITG56118_s_at
	AtNST3/AtSND1	At1g32770	PvSWN2B	AP13ITG56117RC_at
**VND**	AtVND1	At2g18060	PvSWN3A	AP13ITG64965-RC_at
	AtVND2	At4g36160	PvSWN3B	AP13ITG67714_at
	AtVND3	At5g66300	PvSWN6A	AP13CTG29359_s_at
	AtVND4	At1g12260	PvSWN7B	AlamoCTG00660_at
	AtVND5	At1g62700		
	AtVND6	At5g62380		
	AtVND7	At1g71930		
**MYB4**	AtMYB4	At4g38620	PvMYB4	AP13ITG63786_at
**MYB46/83**	AtMYB46	At5g12870	PvMYB46A	KanlowCTG44101_s_at
	AtMYB83	At3g08500	PvMYB46B	AP13ITG55479_s_at
**MYB58/63**	AtMYB58	At1g16490	PvMYB58/63A	AP13ITG56055_at
	AtMYB63	At1g79180	PvMYB58/63C	AP13ITG57153RC_at
			PvMYB58/63D	AP13ITG57154RC_at
**MYB42/85**	AtMYB42	At4g12350	PvMYB42/85A	AP13CTG22878_at
	AtMYB85	At4g22680	PvMYB42/85B	AP13ITG65795_at
			PvMYB42/85D	AP13CTG17231_at

Supplementary Table 4. Numbers of differentially expressed genes in RNA-seq analysis of PvMYB4OX, PvMYB58/63OX, PvMYB42/85OX and PvWRKY12DR switchgrass lines.

		Tiller (compared with the control)		Internode (compared with the control)	
TF Sample ID	Description	Up	Down	Up	Down
WRKY12DR-21	PvWRKY12-DR mild line	582	2346	700	1814
WRKY12DR-33	PvWRKY12-DR strong line	818	2379	753	2340
MYB58/63OX-13	PvMYB58/63A-OX mild line	2736	2410	2873	2598
MYB58/63OX-3	PvMYB58/63A-OX strong line	3338	4146	6090	5303
MYB4OX2/85OX-11	PvMYB42/85A-OX mild line	1475	1895	778	625
MYB4OX2/85OX-29	PvMYB42/85A-OX strong line	1064	1871	1140	2218
MYB4OX-L9	PvMYB4-OX weak line	5290	7395	7577	7112
MYB4OX-L8	PvMYB4-OX mild line	4617	6857	4607	5052
MYB4OX-L6	PvMYB4-OX strong line	5830	6679	5156	5956

Supplementary Table 5. Expression of cell wall-related genes in all switchgrass transgenic lines.

		Internode (log2/Control)									Tiller (log2/Control)								
		MYB4OX			MYB58/63OX		MYB42/85OX		WRKY12DR		MYB4OX			MYB58/63OX		MYB42/85OX		WRKY12DR	
gene_id	Annotation	L6	L8	L9	L13	L3	L11	L29	L21	L33	L6	L8	L9	L13	L3	L11	L29	L21	L33
Lignin biosynthesis genes																			
Pavir.Aa01274	PAL	-2.92				2.48					-0.93				2.31				
Pavir.Fa00937	CCR	-2.62		0.73	0.94	1.56		0.73			-2.08			1.38	1.54		2.64		-0.72
Pavir.Fa01395	4CL	-3.54	-0.95		0.85	2.50					-2.69	-1.42	-1.06		2.29		1.48		
Pavir.Fa01907	COMT	-2.46		2.12		1.64					-0.75			0.70	1.58		1.02		
Pavir.Gb01310	HCT	-3.43	-0.72		1.18	2.49		0.90	0.79		-3.12	-1.48			1.61		1.75		
Pavir.Ia01427	F5H	-2.37		0.61	0.86	0.98					-0.95			0.79	0.95		1.32		
Pavir.J02261	C3H	-3.07		0.89							-1.43					0.94			
Pavir.J12784	CAD	-1.89		0.94		2.39					-1.27		0.91		1.79		2.29		
Pavir.J14845	CCoAOMT	-2.39		1.07		2.07		-0.78			-1.39				1.78				
Pavir.J40960	C4H	-2.14			-0.72	1.90					-1.12				1.48		0.95		
Flavonoid biosynthesis genes																			
Pavir.Ha01439	CHS	-2.43	1.37	2.41	3.79	5.16	2.57		-1.43			1.45		3.66	4.09			-0.80	
Pavir.Ia00185	CHI	-2.10	1.58	2.34		3.38			-1.05			1.10		1.81	2.77			-1.12	
Pavir.Ib04506	CHI	-3.41	0.95	1.61		2.33						1.08		1.55	2.46				
Pavir.J00252	FLS	-4.52	-1.06	-2.42		2.87						1.05		2.22	2.74	-1.44	-1.72		
Pavir.J24485	FLS	-3.88	-1.80	-3.39															
Pavir.Ia04117	TT7	-2.68		0.65		1.54				0.75					1.55		1.57		
Pavir.Ib02986	TT7	-3.33		1.72	1.22	2.52		-1.17	-1.12					1.62	2.47				
Pavir.J09001	TT7	-3.59		1.40	1.00	2.07			-1.12		-0.93			1.94	2.27				
Pavir.Ea02412	DFR			-3.48	3.21	3.73	1.65	2.01				2.00	1.34	3.36	2.47		-1.25		
Pavir.Ga00140	F3H	-2.73		-3.73	2.00	3.43						1.75							
Cellulose and hemi-cellulose biosynthesis genes																			
Pavir.Ca01073	CESA1	-1.53		1.10				-1.35			-0.63			-0.86					
Pavir.Ba01088	CESA3	-1.91		0.92				-1.37			-0.77		-1.04				-1.10		
Pavir.J27681	CESA3	-2.09		1.31															
Pavir.Ba03256	CESA8	-1.72		1.28		1.42		-1.65			-0.73						-1.23	-0.93	
Pavir.J35010	CESA8	-1.34									-1.21	-1.05	-1.12						
Pavir.Eb03139	CESA4	-2.88				3.41		-1.62			-0.90		-1.19	1.66	2.20				
Pavir.J30974	CESA4	-2.77		1.38	1.80	3.53		-1.56	-0.96		-0.93			1.79	2.19			-0.80	
Pavir.Ib00804	CESA7	-2.36		1.56	1.14	3.33		-1.77	-2.15	-1.38		0.92	0.69	1.09	2.32			-1.92	-1.03
Pavir.J07988	CESA7	-2.26		1.68	1.12	3.34		-2.05	-1.23	-0.74				0.95	2.45			-0.86	
Pavir.J21370	CESA7	-2.30	0.75	1.80	1.33	3.67		-2.13	-1.19			0.71		1.35	2.46			-0.80	
Pavir.Bb02205	CESA9	-2.51		1.52	1.35	3.38		-1.75	-1.18					1.50	2.36			-0.85	
Pavir.Ia00526	COB	-1.41		0.79		2.07		-1.31			-0.57				0.83		-1.19	-0.95	
Pavir.J08834	COB	-1.71				1.58		-0.76			-0.61		-0.65		0.69				
Pavir.Ia00651	IRX2	-1.90		1.44		1.98		-1.44			-0.58				1.00				
Pavir.Ib04298	IRX2	-1.88		1.43		1.90		-1.45							0.96				
Pavir.Ia03007	IRX6	-3.70		1.35	2.97	5.43		-2.46	-1.56		-1.07			3.04	4.72				

Pavir.Ib02048	IRX6	-2.16	0.98	2.25	1.91	3.84		-2.27	-0.97			0.74		1.64	2.57				
Secondary wall-related transcription factors																			
Pavir.J16675	MYB4	7.35	5.64				-1.83	0.97			5.13	3.66					1.36		1.26
Pavir.Gb00587	MYB58/63	-2.44		1.81		7.83								1.71	6.30				
Pavir.Ga00752	MYB58/63	-2.70		2.01				-1.60											
Pavir.Aa01159	MYB58/63	-1.37	0.91	1.25		1.61		-2.99	-1.31										
Pavir.J10932	MYB58/63	-2.99															-inf		
Pavir.Ba01239	MYB42/85	-2.80				0.85		2.47	-1.02		-1.30				1.23		4.11		
Pavir.Bb02654	MYB42/85	-2.08				1.01				0.89				1.22	1.37	-1.17	2.25		
Pavir.Fb02321	MYB42/85	-1.95				3.33		-1.45			-1.56	-1.16	-1.32		1.81				
Pavir.J36915	MYB42/85	-1.77	0.65	0.88	1.70	3.23		-1.48						1.29	2.38				
Pavir.Ca02370	MYB46B	-3.61		2.47		3.66		-2.07	-1.58	-1.43	-1.27				2.17			-1.70	
Pavir.Ba01213	MYB32	-2.44									-1.26						1.18		
Pavir.Bb02469	MYB32	-2.09		1.09													1.18		
Pavir.Ha00871	MYB32	-3.97				3.45									2.56				
Pavir.Hb01732	MYB32	-3.46				2.77									2.89		1.94		
Pavir.Fb00364	MYB103	-3.45		1.09		2.38		-2.49	-2.37	-1.11				1.46	2.13			-2.63	-1.23
Pavir.J29561	MYB103	-2.93		1.70		2.41	-1.14	-3.04	-1.81					1.44	2.99			-1.43	
Pavir.Ga00648	WRKY12							-1.33	3.31	3.98	-1.84							2.89	4.49
Pavir.J07835	SWN1	-2.34		1.55	1.64	2.86	-1.12	-1.23	-1.46										
Pavir.J20698	SWN2A	-1.92		1.78		2.37			-2.08										

		internode				tiller				coexpression				
gene_id	Annotation	MYB4-OX	MYB58/63A-OX	MYB42/85A-OX	WRKY12-DR	MYB4-OX	MYB58/63A-OX	MYB42/85A-OX	WRKY12-DR	MYB4	MYB58/63A	MYB42/85A	MYB42/85B	WRKY12
Lignin biosynthesis genes														
Pavir.Aa01274	PAL	-2.92	2.48			-0.93	2.31				Y	Y	Y	
Pavir.Fa00937	CCR	-2.62	1.56	0.73		-2.08	1.54	2.64	-0.72		Y	Y	Y	Y
Pavir.Fa01395	4CL	-3.54	2.50			-2.69	2.29	1.48			Y	Y	Y	
Pavir.Fa01907	COMT	-2.46	1.64			-0.75	1.58	1.02				Y	Y	
Pavir.Gb01310	HCT	-3.43	2.49	0.90		-3.12	1.61	1.75			Y	Y	Y	
Pavir.Ia01427	F5H	-2.37	0.98			-0.95	0.95	1.32			Y	Y		
Pavir.J02261	C3H	-3.07				-1.43					Y		Y	
Pavir.J12784	CAD	-1.89	2.39			-1.27	1.79	2.29			Y		Y	
Pavir.J14845	CCoAOMT	-2.39	2.07	-0.78		-1.39	1.78				Y	Y	Y	
Pavir.J40960	C4H	-2.14	1.90			-1.12	1.48	0.95						
Flavonoid biosynthesis genes														
Pavir.Ha01439	CHS	-2.43	5.16				4.09				Y	Y	Y	Y
Pavir.Ia00185	CHI	-2.10	3.38				2.77				Y	Y	Y	
Pavir.Ib04506	CHI	-3.41	2.33				2.46					Y		
Pavir.J00252	FLS	-4.52	2.87				2.74	-1.72		Y				
Pavir.J24485	FLS	-3.88												
Pavir.Ia04117	TT7	-2.68	1.54		0.75		1.55	1.57			Y	Y	Y	
Pavir.Ib02986	TT7	-3.33	2.52	-1.17			2.47				Y	Y	Y	
Pavir.J09001	TT7	-3.59	2.07			-0.93	2.27							
Pavir.Ea02412	DFR		3.73	2.01			2.47	-1.25						
Pavir.Ga00140	F3H	-2.73	3.43							Y				
Cellulose biosynthesis genes (primary wall)														
Pavir.Ca01073	CESA1	-1.53		-1.35		-0.63					Y			
Pavir.Ba01088	CESA3	-1.91		-1.37		-0.77		-1.10			Y		Y	
Pavir.J27681	CESA3	-2.09									Y	Y	Y	Y
Pavir.Ba03256	CESA8	-1.72	1.42	-1.65		-0.73		-1.23						
Pavir.J35010	CESA8	-1.34				-1.21								
Cellulose biosynthesis genes (secondary wall)														
Pavir.Eb03139	CESA4	-2.88	3.41	-1.62		-0.90	2.20				Y	Y	Y	
Pavir.J30974	CESA4	-2.77	3.53	-1.56		-0.93	2.19				Y	Y	Y	
Pavir.Ib00804	CESA7	-2.36	3.33	-1.77	-1.38		2.32		-1.03	Y				
Pavir.J07988	CESA7	-2.26	3.34	-2.05	-0.74		2.45				Y			
Pavir.J21370	CESA7	-2.30	3.67	-2.13			2.46							
Pavir.Bb02205	CESA9	-2.51	3.38	-1.75			2.36				Y	Y	Y	
Other cellulose and hemi-cellulose biosynthesis genes														
Pavir.Ia00526	COB	-1.41	2.07	-1.31		-0.57	0.83	-1.19						
Pavir.J08834	COB	-1.71	1.58	-0.76		-0.61	0.69							
Pavir.Ia00651	IRX2	-1.90	1.98	-1.44		-0.58	1.00				Y	Y	Y	
Pavir.Ib04298	IRX2	-1.88	1.90	-1.45			0.96			Y	Y	Y	Y	Y
Pavir.Ia03007	IRX6	-3.70	5.43	-2.46		-1.07	4.72							
Pavir.Ib02048	IRX6	-2.16	3.84	-2.27			2.57							
Secondary wall-related transcription factors														
Pavir.J16675	MYB4	7.35		0.97		5.13		1.36	1.26	Y				
Pavir.Gb00587	MYB58/63	-2.44	7.83				6.30				Y	Y	Y	
Pavir.Ga00752	MYB58/63	-2.70		-1.60							Y	Y	Y	
Pavir.Aa01159	MYB58/63	-1.37	1.61	-2.99							Y	Y	Y	
Pavir.J10932	MYB58/63	-2.99						-inf						
Pavir.Ba01239	MYB42/85	-2.80	0.85	2.47		-1.30	1.23	4.11			Y	Y	Y	Y
Pavir.Bb02654	MYB42/85	-2.08	1.01		0.89		1.37	2.25			Y	Y	Y	
Pavir.Fb02321	MYB42/85	-1.95	3.33	-1.45		-1.56	1.81				Y	Y	Y	
Pavir.J36915	MYB42/85	-1.77	3.23	-1.48			2.38				Y	Y	Y	
Pavir.Ca02370	MYB46B	-3.61	3.66	-2.07	-1.43	-1.27	2.17				Y	Y	Y	Y
Pavir.Ba01213	MYB32	-2.44				-1.26		1.18					Y	
Pavir.Bb02469	MYB32	-2.09						1.18		Y	Y	Y	Y	
Pavir.Ha00871	MYB32	-3.97	3.45				2.56				Y			
Pavir.Hb01732	MYB32	-3.46	2.77				2.89	1.94			Y	Y	Y	
Pavir.Fb00364	MYB103	-3.45	2.38	-2.49	-1.11		2.13		-1.23		Y	Y	Y	
Pavir.J29561	MYB103	-2.93	2.41	-3.04			2.99				Y	Y	Y	Y
Pavir.Ga00648	WRKY12			-1.33	3.98	-1.84			4.49					
Pavir.J07835	SWN1	-2.34	2.86	-1.23								Y	Y	Y
Pavir.J20698	SWN2A	-1.92	2.37								Y	Y	Y	Y
Pavir.Eb02718	SND2	-2.81	2.45	-1.76			2.00				Y	Y	Y	
Pavir.J05241	SND2	-2.41			-0.81		1.72				Y	Y	Y	

Supplementary Table 6. Differential expression of secondary cell wall-related genes in tillers and internodes of PvMYB4OX, PvMYB58/63OX, PvMYB42/85OX and PvWRKY12DR transgenic switchgrass lines compared with control. Values significantly different from control (adjusted P ≤ 0.05) are presented as log2 fold-change in expression compared to control. The right column indicates the existence of a correlation relationship (Y) identified by co-expression analysis.

Supplementary Table 7. Sequences for the gene-specific primers used in this work.

Gene	usage	Forward Sequences	Reverse sequences
C4H	qRT-PCR	GGGCAGTTCAGCAACCAGAT	CGCGTTTCCGGGACTCTAG
4CL	qRT-PCR	CGAGCAGATCATGAAAGGTTACC	CAGCCAGCCGTCCTTGTC
CCR	qRT-PCR	GCGTCGTGGCTCGTCAA	TCGGGTCATCTGGGTTCCT
HCT	qRT-PCR	GCAGAAGGAGCAGCAGTCATC	CGAGCGGCAATAGTCGTTGT
C3H	qRT-PCR	TTGAGATGGTTGTGTCCGCTTA	AGGCGGTCCCTTCTCTCATT
CCoAMOT	qRT-PCR	CCGTCTTTCTTTTTTTGGCTCTT	GCATGAAAATGATGACAGTTTCCA
COMT	qRT-PCR	CAACCGCGTGTTCAACGA	CGGTGTAGAACTCGAGCAGCTT
CAD	qRT-PCR	TCACATCAAGCATCCACCATCT	GTTCTCGTGTCCGAGGTGTGT
F5H	qRT-PCR	CTCTTCTATTTGTGCGTGTAACTGTGT	CAGCCCTATAGCATCGACATGA
PAL	qRT-PCR	CATATAGTGTGCGTGCGTGTGT	CTGGCCCGCCAATCG
Ubi	qRT-PCR	TTCGTGGTGGCCAGTAAG	AGAGACCAGAAGACCCAGGTACAG
MYB58/63A	qRT-PCR	GCCTGGAGGAGCTCGAGAT	CAACAAGCATGTCGAAGTCGAA
MYB58/63C	qRT-PCR	GGGCCCAGTCTCTACCTACTT	AGGAGTTGTTCGGTTGCAG
MYB42/85A	qRT-PCR	CTCGGCAACAGATGGTCAAA	AGTGGTTCTTGATCTCGTTGTCAGT
MYB42/85C	qRT-PCR	TTCAACACTTCCCAGCTCAG	ACTTCGAGGAGAAAGCTCCA
WRKY12	qRT-PCR	ACGGCCAGAAGGTTGTCAAG	TGGGTGCACCGGTAGTAGCT
SWN1	qRT-PCR	AACAACGTGTAGCCCAGACA	CGCGTGTAGATGGAGTTAGC
SWN2	qRT-PCR	ACAAGGAGTCGGGAGGCG	CGCAGCCTTGCCACCATC
SND2A	qRT-PCR	GTCCCTGACCAAGATGTGC	TTATCTTCCTGCCTTTCTCTCC
SND2B	qRT-PCR	TGAGTTACAAGAAGGGATCCAA	TCCAGCTACCCACTGAACAC
SND2C	qRT-PCR	ATTTAGGAGCAGCTCATGCC	TGTTTCTTCACGTGTGAGGTAGT
SND2D	qRT-PCR	AGGGATTTAGGAGCAGGTCA	TTCGCGTGTGAGGTAGGTAG
MYB4	qRT-PCR	CTCAATCTCGACCTCTGCATCA	ACGAGCTCCTGGTCCTCTTCT
MYB46	qRT-PCR	CTGGAGGAGCTGATGAAAGA	CCCTCCCAATCATTCAACTT
MYB103	qRT-PCR	CCAAGAACTAACACCGTCCA	TGGTCATTTGGTCCCATAAA
CESA4	qRT-PCR	CTCCTCTGGGTCAAGATCG	TGACTGTGTTGCAGTTGGTG
CESA9	qRT-PCR	CAAGCAGTGTGGCATCAACT	TATGCATGCACAAGCTCCA
WRKY12	In Situ Hybridization	TCTGCAGATGCCGTTGTT	CGCTCCACTCGCTTCTTC
	In Situ Hybridization (T7 primer)	GCGTAATACGACTCACTATAGGGTCTGCAGATGCCGTTGTT	GCGTAATACGACTCACTATAGGGCGCTCCACTCGCTTCTTC
MYB42/85A	In Situ Hybridization	GATTCAACCAAGTCCGACGA	GATCATGTAGCCATCGACCAA

	In Situ Hybridization (T7 primer)	GCGTAATACGACTCACTATAGGGGATTCAACCAAGTCCGACGA	GCGTAATACGACTCACTATAGGGGATCATGTAGCCATCGACCAA
MYB42/85C	In Situ Hybridization	CAACTCCAAGGCGTCTACTT	ATCTGAGCTGGGAAGTGTTG
	In Situ Hybridization (T7 primer)	GCGTAATACGACTCACTATAGGGCAACTCCAAGGCGTCTACTT	GCGTAATACGACTCACTATAGGGATCTGAGCTGGGAAGTGTTG
MYB58/63A	In Situ Hybridization	CGGCTCCAAGAAGAAGAAGAA	CTCGTTGCAACAGCAGAGTA
	In Situ Hybridization (T7 primer)	GCGTAATACGACTCACTATAGGGCGGCTCCAAGAAGAAGAAGAA	GCGTAATACGACTCACTATAGGGCTCGTTGCAACAGCAGAGTA
MYB58/63C	In Situ Hybridization	CGAGATCAAGAACGTGTGGAA	TTCTCCAACCACCAATCGTC
	In Situ Hybridization (T7 primer)	GCGTAATACGACTCACTATAGGGCGAGATCAAGAACGTGTGGAA	GCGTAATACGACTCACTATAGGGTTCTCCAACCACCAATCGTC
MYB58/63A	overepxression construction	caccATGGGCAAGGGCCGGGCAC	TCAGAAACCCATCTGGTTACT
MYB58/63A	RNAi construction	CGGTGTCGTGCTACTTCCA	TATACGACGGCGATGAGTCC
MYB42/85A	overepxression construction	caccATGGGGCGGCAGCCGTGC	TTAATTTGCTCCGTTTGAGC
MYB42/85A	RNAi construction	GATGGGCATCGACCCGGTC	TTAATTTGCTCCGTTTGAGC
SWN2	RNAi construction	GACGACCTCCTCCACGGCG	CACAGGTCGTCGTCGCTGG
SND2	RNAi construction	GGAGAAGACCAACTGGGTG	GCACACTTGCACATCTTGGT
F5H	promoter cloning for transactivation	caccCACAGCCGCCCATAA	GATCGGCAACACCTTTTTGT
COMT	promoter cloning for transactivation	caccGGAGCCAAACACCTAC	GGCTGATGGCTGGGCTGG
MYB4	gene cloning for transactivation	caccATGGGGCGGTCGCC	TCACTTCATCTCGAGGCCTC
MYB46	gene cloning for transactivation	caccATGAGGAAGCCGGA	TCATTCAACTTGGAAATCAAGG
MYB58/63A	gene cloning for transactivation	caccATGGGGAAGGGCCGG	TCAGAAACCCATCTGGTTACT
MYB42/85A	gene cloning for transactivation	caccATGGGGCGGCAGCC	TTAATTTGCTCCGTTTGAGC

SUPPLEMENTARY METHODS

Definition of BF score

To detect co-expression relationships among genes in transcriptome data, a new co-expression evaluation score, called “BF score” (Biclustering-Fundamental score), was developed in this paper. It is used to quantitatively evaluate the co-expression level of a gene pair. A two-step procedure is designed to calculate the “BF score” from the input gene expression matrix. The first step is to select effective conditions for a pair of genes. The second step is to calculate the Pearson Correlation Coefficient (PCC) of the two genes under the selected effective conditions in Step 1.

With these two steps, the removal of ineffective conditions will enhance the sensitivity to find co-expressed gene pairs under specific sets of conditions. Below is an example from the switchgrass transcriptome dataset. Our method shows an increased PCC value between two genes under local conditions compared with that under global conditions.

Probe_id	CK_7_AC1	CK_8_AC3	CK_9_AC5	Crown_E4-1	Crown_E4-2	Crown_E4-3	I3MiddleVB_E4-1	I3MiddleVB_E4-2	I3MiddleVB_E4-3	I3Middle_E4-1	I3Middle_E4-2	I3Middle_E4-3	---
AP13CTG16431_at	9.88	9.64	9.31	8.51	8.86	8.49	9.14	9.14	9.62	10.17	10.58	10.33	---
AP13CTG23789_at	9.51	7.8	8.82	7.7	7.74	7.72	10.38	10.41	9.52	8.66	8.23	8.76	---

PCC(x,y)=0.4340076

Probe_id	CK_7_AC1	CK_9_AC5	I3MiddleVB_E4-2	I3MiddleVB_E4-3	I3Middle_E4-1	I3Middle_E4-3	I4Middle_E4-1	LeafBlade_E4-1	LeafBlade_E4-2	LeafBlade_E4-3	Root_E4-3	SeedGermination_24h-1	...
AP13CTG16431_at	9.88	9.31	9.14	9.62	10.17	10.33	10.31	4.98	5.3	5.47	9.13	7.11	---
AP13CTG23789_at	9.51	8.82	10.41	9.52	8.66	8.76	8.92	7.1	6.97	7.26	8.64	7.13	---

PCC(x,y)=0.7355932

Figure 1: An example of PCC value between two genes under global conditions and under selected conditions. In this example from the switchgrass dataset, matrix columns and rows represent multiple conditions and two gene probes, respectively. The gene expression value under each condition was normalized by the Robust Multi-array Average (RMA) method. The PCC value between the two genes is 0.43 under all conditions. After application of the bi-clustering method

"QUBIC" (Li *et al.*, 2009; Zhang *et al.*, 2017) to select effective conditions (marked in green), the PCC value between the two genes increased to 0.74 under selected conditions.

Step 1. Selection of subset conditions

A bi-clustering method "QUBIC" (Li *et al.*, 2009; Zhang *et al.*, 2017) is utilized to select effective conditions in the transcriptome data matrix. For a given set of genes G, QUBIC is designed to identify maximal subsets of conditions C (called "effective conditions") under which the set of genes is significantly grouped in the same bi-cluster (Li *et al.*, 2009; Zhang *et al.*, 2017).

Step 2. Calculation of BF score

The BF score between two genes g_i and g_j in the matrix dataset with N samples is defined in the following formula:

$$\mathrm{BF}(g_i, g_j) = \left(\sum_{k=1}^{N} I(g_i^k, g_j^k)\,/N\right) \times \mathrm{PCC}(g_i, g_j)^{\text{where } I(g_i^k, g_j^k)=1}$$

Where $I(g_i^k, g_j^k)$ is an indication score that is equal to 1 when g_i and g_j are in the same bi-cluster under k^{th} condition. PCC(x, y) represents Pearson correlation value.

Step 3. Cut-off threshold of BF score

To determine the threshold of BF score, we examined the distribution of BF scores of all gene pairs from 100 datasets.

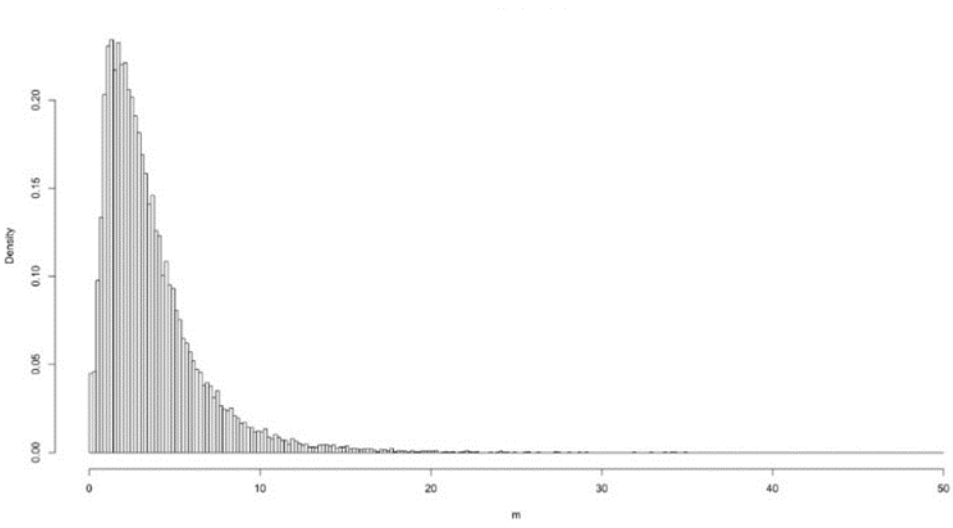

Figure 2. The distribution of BF scores of all gene pairs in 100 datasets.

The P-value of BF score for each gene pair is defined in the following formula.

$$P(g_i, g_j) = \frac{Rank(g_i, g_j)}{M}$$

$Rank(g_i, g_j)$ is the rank of the $F(g_i, g_j)$ scores for gene g_i across genes g_j; M is the total number of genes in the matrix. Gene pairs with P-value ≤ 0.05 in BF score were defined to be significantly co-expressed.

Comparison of the bi-clustering method with the standard Pearson correlation

Pearson correlation (PCC) for gene pairs under global conditions is the most popular method for co-expression analysis (Serin et al., 2016). The PCC method has been used to identify candidate TFs involved in cell wall formation in Arabidopsis and rice (Ruprecht et al., 2011; Hirano et al., 2013). However, strongly correlated expression of genes can occur under rather specific (and perhaps still to-be-identified) conditions (Li et al., 2009). For example, a TF may regulate its target gene in specific tissues or in a particular tissue only under specific treatments; this leads to the expression levels of the TF and its target gene showing strong co-expression under certain conditions, but weakly or non- correlated expression under other conditions. Our method based

on the bi-clustering algorithm (Li et al., 2009; Zhang et al., 2017) increases sensitivity to detect gene pairs that show strongly correlated expression under specific sets of conditions.

To compare the effectiveness and read-out of our bi-clustering method with standard PPCC analysis, we calculated the Pearson correlation coefficient (PCC) value and BF value between F5H and all TFs in microarray datasets (93 samples, Zhang et al., 2013) (Supplementary Dataset 7). Using the PCC method, we set a PCC threshold of 0.7 and identified 42 TFs co-expressed with F5H. In contrast, using the bi-clustering method, 236 TFs were identified to be co-expressed with F5H. Among them, 32 TFs were detected as co-expressors using both methods, whereas 204 and 10 TFs were identified as co-expressors by bi-clustering and PCC methods, respectively. This shows a higher sensitivity to detect co-expressed gene pairs using the bi-clustering method than using the PCC method, especially for those genes strongly co-expressed under certain conditions. For example, the gene pair AP13CTG29842_at and AP13ITG56842_at (F5H) shows a very high BF score (57.77, defined as "significantly correlated") using the bi-clustering method, but a low PCC value (0.56, defined as "not correlated") using the PCC method. This is because a high correlation for expression of this gene pair occurs in stem tissues, but not in seed tissues (Supplementary Dataset 7).
